# Planar Chiral [2.2]Paracyclophane-Based Bisoxazoline Ligands and Their Applications in Cu-Mediated N–H Insertion Reaction

**DOI:** 10.3390/molecules24224122

**Published:** 2019-11-14

**Authors:** Daniel M. Knoll, Yuling Hu, Zahid Hassan, Martin Nieger, Stefan Bräse

**Affiliations:** 1 Institute of Organic Chemistry (IOC), Karlsruhe Institute of Technology (KIT), Fritz-Haber-Weg 6, 76131 Karlsruhe, Germany; daniel.knoll@kit.edu (D.M.K.); yuling.hu0311@hotmail.com (Y.H.); zahid.hassan@kit.edu (Z.H.); 2 Department of Chemistry, University of Helsinki, P.O. Box 55 A.I. Virtasen aukio 1, 00014 Helsinki, Finland; martin.nieger@helsinki.fi; 3 Institute of Toxicology and Genetics (ITG), Karlsruhe Institute of Technology (KIT), Hermann-von-Helmholtz-Platz 1, D-76344 Eggenstein-Leopoldshafen, Germany

**Keywords:** [2.2]paracyclophane ligands, N–H insertion, α-diazocarbonyls, planar chirality, copper catalysis

## Abstract

New catalysts for important C–N bond formation are highly sought after. In this work, we demonstrate the synthesis and viability of a new class of planar chiral [2.2]paracyclophane-based bisoxazoline (BOX) ligands for the copper-catalyzed N–H insertion of α-diazocarbonyls into anilines. The reaction features a wide substrate scope and moderate to excellent yields, and delivers the valuable products at ambient conditions.

## 1. Introduction

**Figure 1:**
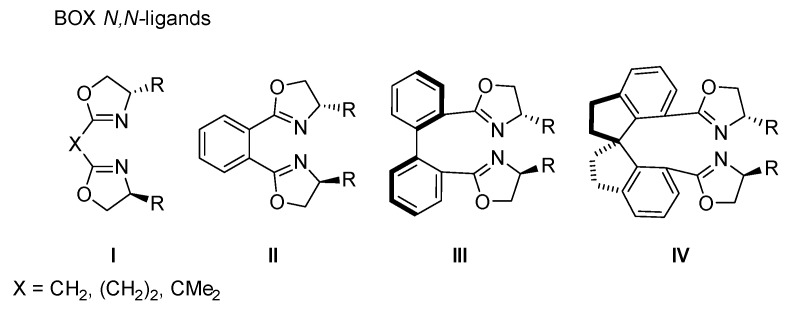
Representative classes of bisoxazoline-containing *N,N*-ligand systems used in asymmetric catalysis.

The copper-catalyzed N–H insertion of carbenoids such as easily prepared α-diazocarbonyls is a powerful method for the preparation of highly valuable bioactive molecules and pharmaceutical products [1]. In the past decade, various enantioselective chelating ligands based on the bis(oxazoline) motifs have been established for this transformation (Figure 1) [2,3,4,5,6,7,8].

**Figure 2:**
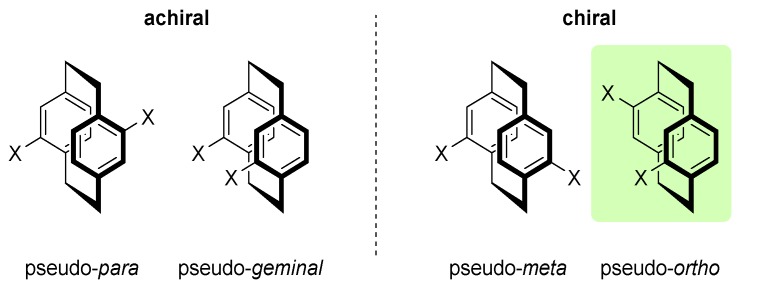
Achiral and chiral isomers of homodisubstituted PCP with the substituents on different decks. The pseudo-*ortho* isomer is the most suitable candidate for PCPBOX ligands.

While SpiroBOX **IV** (Figure 1) combines axial chirality from the spiro backbone with the central chirality of the 2-substituted BOX moiety, [2.2]paracyclophane (PCP) exhibits planar chirality, which has previously demonstrated remarkable performance as planar chiral ligand or chiral catalyst in asymmetric catalysis [9]. Notable examples include the addition of alkyl, aryl, alkynyl, and alkenyl zinc reagents to aromatic and aliphatic aldehydes and imines that are catalyzed by PCP ligands[10,11,12,13,14]. The PCP core allows different substituents to be positioned regioselectively using carefully chosen reaction parameters [15]. PCP displays planar chirality if only one substituent is introduced to one of the two aromatic benzene rings (decks). If the other deck is substituted as well, especially with both substituents being identical, only the pseudo-*ortho* and pseudo-*meta* PCP exhibit chirality, as the other two PCP isomers show higher symmetry (Figure 2) [15]. Thus, the pseudo-*ortho* PCP isomer is the most suitable for a chelating BOX ligand with PCP as the backbone – referred to here as [2.2]paracyclophane-based bisoxazoline (PCPBOX).

**Scheme 1:**
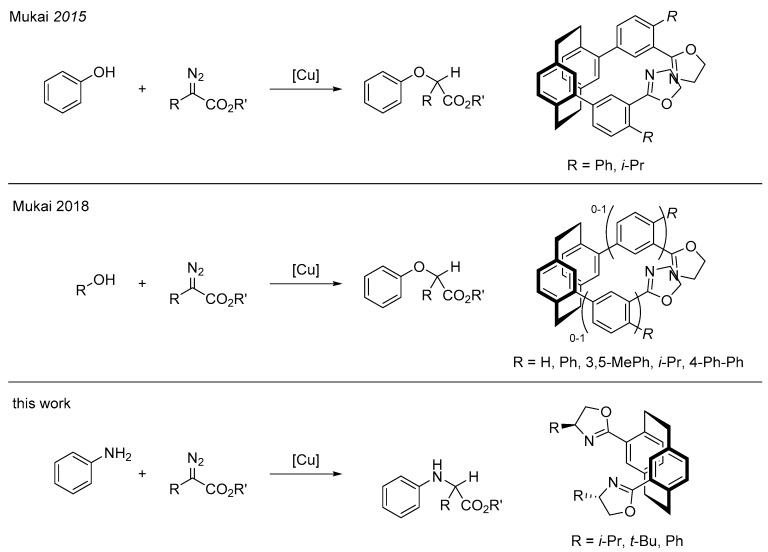
The earlier work of Mukai et al. on copper-catalyzed O–H insertion reaction and this work on N–H insertion reaction.

Mukai et al. recently reported on PCPphBOX that employed phenyl spacers bearing a sterically demanding substituent between PCP and BOX (Scheme 1). These PCPphBOX served as promising chiral ligands for the asymmetric copper-catalyzed inter- and intra-molecular aromatic O–H insertion reaction with up to 80%*ee* [16,17]. Mukai et al. additionally investigated PCPBOX ligands, with phenyl, biphenyl, and without the phenyl as spacer groups for comparative studies. We thus set out to explore the PCPBOX ligands in copper-catalyzed N–H insertion to expand on their versatility.

## 2. Results

### 2.1. Synthesis

**Scheme 2:**
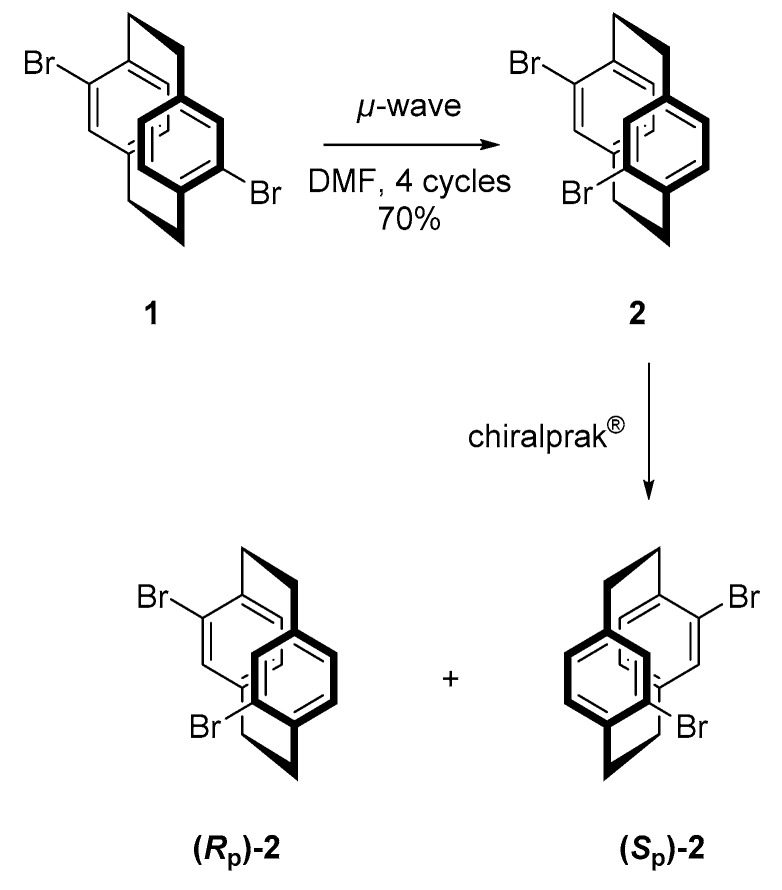
Preparation of enantiopure pseudo-*ortho* PCP dibromide **2** via microwave-assisted isomerization.

Access to pseudo-*ortho* disubstituted PCPs leads through the thermal or microwave-assisted isomerization of the easily accessible pseudo-*para* dibromide of PCP [18]. In this way, pseudo-*ortho* dibromide **2** was obtained in 70% yield. On this stage, chromatographic separation of the racemic **2** was achieved via a Chiralprak^®^ AZ-H column (Scheme 2).

**Scheme 3:**
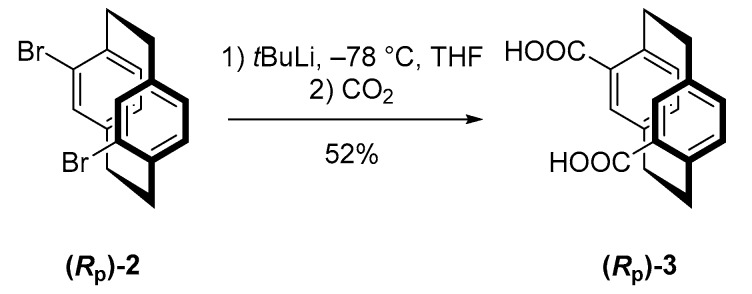
Conversion of the dibromo PCP to the dicarboxylic acid **3**.

The obtained (*R*_p_)-**2** and (*S*_p_)-**2** were subjected to a two-step lithiation-carboxylation procedure to afford the enantiopure carboxylic acids **3** (Scheme 3). However, while the conversion of (*R*_p_)-**2** smoothly delivered (*R*_p_)-**3** in good yield, the conversion of (*S*_p_)-**2** left us with inconclusive results.

**Scheme 4:**
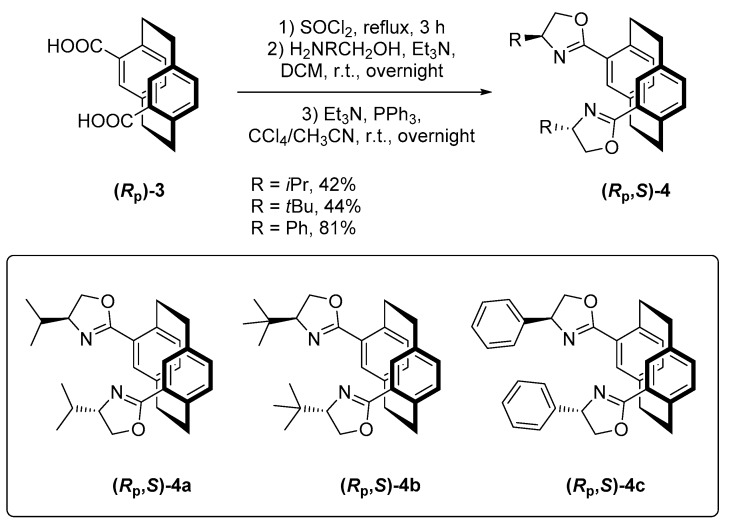
Preparation of enantiopure PCPBOX **4a**–**c**.

With (*R*_p_)-**3** in hand, we proceeded with the PCPBOX synthesis by subjecting it to condensation conditions with suitable amino alcohols to afford the respective hydroxyl amides. Under Appel conditions, cyclization and dehydration is achieved to afford the enantiopure PCPBOXs **4a**–**c** in moderate to good yields (Scheme 4).

### 2.2. Catalysis

The synthesized PCPBOX ligands were tested in the copper-catalyzed N–H insertion reaction. The catalyst is generated in situ from ligand **4** and a rationally selected copper source. For the optimization of the copper source, the diastereomeric mixture (*S*_p_,*S*)/(*R*_p_,*S*)-**4** was used. The competition between N–H insertion and β-hydride elimination (BHE) leads to a mixture of desired product **7** and the olefinic product **8**.

The initially tested Cu(MeCN)_4_PF_6_ complex shows good selectivity (Table 1, entry 1). Simple copper (I) chloride does not deliver the desired product at all (entry 2). Lowering the temperature to room temperature increased the selectivity to an excellent ratio of 93:5 with Cu(MeCN)_4_PF_6_ (entry 4). When β-hydrogen lacking **6b** was used (entry 7–10), product **9** was detected from the dimerization of the α-diazocarbonyl **6b**. Dropwise addition of **6b** to the reaction mixture alleviated this issue for the most part. With these optimized reaction conditions, the same copper source (Cu(MeCN)_4_PF_6_) leads to excellent yields of 98% for the desired product **7b**. Notably, in both cases the product was formed even in the absence of the ligand **4** in 13% and 40% yield respectively.

**Figure 3:**
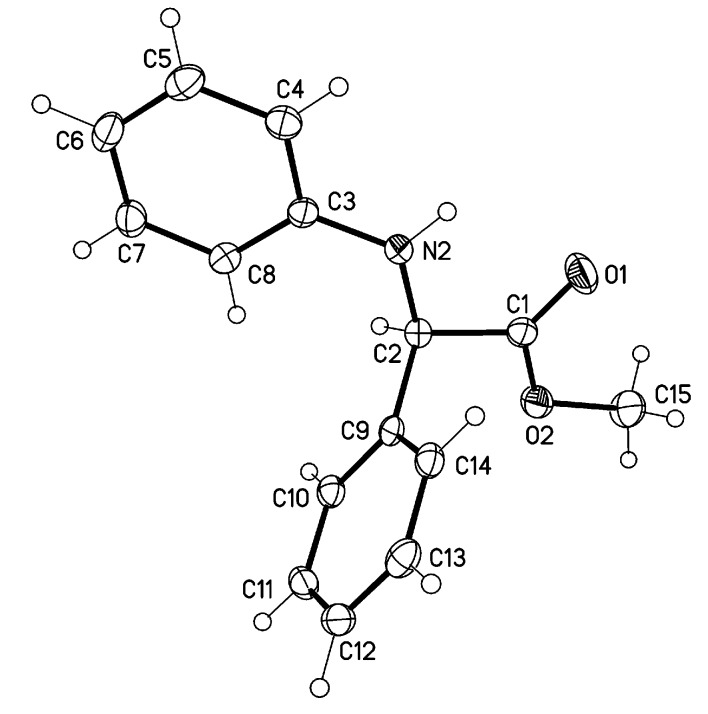
Molecular structure of **7b** (displacement parameters are drawn at 50% probability level).

The molecular structure of the N–H insertion product **7b** was further confirmed unambiguously by single crystal X-Rays structure analysis (Figure 3, for further details see Electronic Supplementary Information and cif-file, CCDC 1962906 (**7b**) contains the supplementary crystallographic data for this paper. These data can be obtained free of charge from The Cambridge Crystallographic Data Centre via www.ccdc.cam.ac.uk/data_request/cif).

A series of other aniline derivatives and α-diazocarbonyls were investigated to further test the substrate scope for this reaction. A wide range of products were obtained in short reaction times and in good to excellent yields (Table 2). While unsubstituted phenyl rings as substituents gave the best yields (entry 2), benzyl substituents also delivered the product in very good yield (entries 3–8). However, a drop in yields was observed employing electron donating groups in the *meta*-position (entry 5). This contrasts with findings of Zhou et al. showing no such drop for similar substitution patterns [2]. If non-aromatic anilines were used, no product formation could be observed (entries 11–12).

We turned our attention towards the enantioselective N–H insertion with unsaturated α-diazocarbonyls. The products of this reaction are valuable intermediates that can be used in the total synthesis of biologically important products such as Rostratin B.-D [19]. The ligands discussed in Scheme 4 were tested with results summarized in Table 3. While only low yields of 22% were achieved with 5 mol% (*R_p_,S*)-**4a** (Table 3, entry 1), this could be increased to 38% by using 10 mol% ligand. Dramatically increased yields were observed for the more sterically demanding (*R_p_,S*)-**4b** and (*R_p_,S*)-**4c** (entry 3–4). All these enantiopure ligands did not induce any considerable enantioselectivity as determined by chiral HPLC. This further verifies the observations obtained by Mukai et al. that the combination of planar and central chirality in PCPBOX ligands suffers from very low enantioinduction [17].

## 3. Materials and Methods

Benzyl 2-diazopropanoate [20], Methyl 2-diazo-2-phenylacetate [21] and *tert*-Butyl 2-diazopropanoate [20] were prepared according to literature procedures.

*4,16-Dibromo[2.2]paracyclophane (**1**)*

A solution of Br_2_ (5.50 mL, 17.0 g, 106 mmol, 2.20 equiv.) in CH_2_Cl_2_ (50 mL) was prepared. A suspension of iron powder (0.14 g, 2.4 mmol, 0.05 equiv.) in 6.25 mL of the Br_2_/CH_2_Cl_2_ solution was diluted in 50 mL of CH_2_Cl_2_ and stirred at room temperature for 1 h. The solution was then brought to reflux for 2 h. CH_2_Cl_2_ (50 mL) and [2.2]paracyclophane (10.0 g, 48.0 mmol, 1.00 equiv.) were added to the mixture subsequently. After the remaining bromine solution was added dropwise over a period of 4 h, the mixture was stirred at room temperature for 3 d. Saturated Na_2_S_2_O_3_ solution was added and the reaction mixture was stirred at room temperature until the bromine color disappeared. The organic phase was separated and filtrated, the precipitate was recrystallized from hot toluene to obtain the title product as an off-white solid, 5.40 g, 14.8 mmol, 31%.

^1^H-NMR (400 MHz, CDCl_3_) δ/ppm = 7.14 (dd, *J* = 7.8, 1.8 Hz, 2H, 2 × C_Ar_H), 6.51 (d, *J* = 1.8 Hz, 2H, 2 × C_Ar_H), 6.44 (d, *J* = 7.8 Hz, 2H, 2 × C_Ar_H), 3.50 (ddd, *J* = 12.8, 10.3, 2.0 Hz, 2H, 2 × CH_PC_), 3.16 (ddd, *J* = 12.1, 10.2, 4.6 Hz, 2H, 2 × CH_PC_), 2.95 (ddd, *J* = 12.1, 11.4, 2.0 Hz, 2H, 2 × CH_PC_), 2.85 (ddd, *J* = 13.0, 10.6, 4.6 Hz, 2H, 2 × CH_PC_). ^13^C-NMR (101 MHz, CDCl_3_) δ/ppm = 141.3 (C_q_, 2 × C_Ar_*)*, 138.6 (C_q_, 2 × C_Ar_*)*, 137.4 (+, CH, 2 × C_Ar_*)*, 134.2 (+, CH, 2 × C_Ar_*)*, 128.4 (+, CH, 2 × C_Ar_*)*, 126.8 (C_q_, 2 × C_Ar_-Br), 35.5 (−, 2 × CH_2_), 32.9 51 (−, 2 × CH_2_). IR (ATR): $\tilde{v}$/cm^−1^ = 2932 (vw), 2849 (vw), 1895 (vw), 1583 (vw), 1532 (vw), 1474 (vw), 1449 (vw), 1432 (vw), 1390 (w), 1313 (vw), 1185 (vw), 1104 (vw), 1030 (w), 947 (vw), 899 (w), 839 (w), 855 (w), 830 (w), 706 (w), 669 (w), 647 (w), 522 (vw), 464 (w), 393 (vw). MS (EI, 70 eV), *m*/*z* (%): 364/366/368 (3/6/3) [M]^+^, 184/182 (18/18) [M − C_8_H_7_Br]^+^, 104 (100) [C_8_H_8_]^+^. HRMS (EI, C_16_H_14_^79^Br_2_) calc. 363.9457, found 363.9455.

*(rac)-4,12-Dibromo[2.2]paracyclophane (rac)-**2***

In a 10 mL microwave vessel was placed 4,16-dibromo[2.2]paracyclophane (500 mg, 1.37 mmol, 1.00 equiv.) and DMF (1.00 mL). The device was programmed to heat the mixture to 180 °C with a holding time set as 6 min. The maximum pressure for the system was set at 17.2 bar and the power was set at 300 W. After cooling to room temperature, the mixture was diluted with DMF (2 mL) and the precipitate was collected by filtration. The reaction was repeated under the same conditions until all the starting material (5.00 g, 13.7 mmol, 1.00 equiv.) reacted. The combined filtrate was poured into water (75 mL) and extracted with EtOAc (3 × 100 mL). The combined organic phase was washed with water and brine, dried over Na_2_SO_4_ and concentrated under reduced pressure to give the title product as a pale brown power, 3.50 g, 9.65 mmol, 70%.

*R_f_* = 0.68 (*c*-Hex/EtOAc = 9:1). ^1^H-NMR (400 MHz, CDCl_3_) δ/ppm = 7.22 (d, *J* = 1.6 Hz, 2H, 2 × C_Ar_H), 6.56 (d, *J* = 7.8 Hz, 2H, 2 × C_Ar_H), 6.52 (dd, *J* = 7.9, 1.7 Hz, 2H, 2 × C_Ar_H), 3.47 (ddd, *J* = 13.3, 9.6, 2.2 Hz, 2H, 2 × C*H_PC_*), 3.10 (ddd, *J* = 13.0, 9.6, 6.8 Hz, 2H, 2 × C*H_PC_*), 3.06–2.94 (m, 2H, 2 × C*H_PC_*), 2.82 (ddd, *J* = 13.3, 10.1, 6.9 Hz, 2H, 2 × C*H_PC_*). ^13^C-NMR (101 MHz, CDCl_3_) δ/ppm = 141.3 (C_q_, 2 × *C_Ar_)*, 138.7 (C_q_, 2 × *C_Ar_)*, 135.0 (+, CH, 2 × *C_Ar_)*, 132.7 (+, CH, 2 × *C_Ar_)*, 131.7 (+, CH, 2 × *C_Ar_)*, 126.7 (C_q_, 2 × *C*_Ar_-Br), 35.8 (−, 2 × CH_2_), 32.5 (−, 2 × CH_2_). IR (ATR): $\tilde{v}$/cm^−1^ = 2923 (w), 2848 (w), 1583 (w), 1537 (w), 1474 (w), 1449 (w), 1431 (w), 1391 (m), 1272 (w), 1237 (w), 1201 (w), 1185 (w), 1030 (m), 902 (m), 858 (m), 785 (w), 705 (m), 644 (m), 475 (m). MS (70 eV, EI) *m*/*z* (%): 368/366/364 (22/43/22) [M]^+^, 288/286 (13/12) [M + H – Br]^+^, 184/182 (80/100) [M – C_8_H_6_Br]^+^, 104 (68) [C_8_H_8_]^+^. HRMS (EI, C_16_H_14_^79^Br_2_) calc. 363.9462, found 363.9461.

*(R_p_)-4,12-Dibromo[2.2]paracyclophane (R_p_-**2**) / (S_p_)-4,12-Dibromo[2.2]paracyclophane (S_p_-**2**)*

Separation of (*rac*)-4,12-dibromo[2.2]paracyclophane (**2**) was performed by semi-preparative chiral HPLC. For details see Electronic Supporting Information.

*(R_p_)-4,12-Dicarboxy[2.2]paracyclophane (R_p_-**3**)*

To a solution of (*R*_p_)-4,12-dibromo[2.2]paracyclophane (1.50 g, 4.12 mmol, 1.00 equiv.) in abs. THF (50 mL) was added 9.71 mL of *t*-butyllithium (1.7 M in pentane, 15.4 mmol, 4.00 equiv.) dropwise at −78 °C. After stirring at −78 °C for 3 h, CO_2_ was bubbled through the solution via a long needle under stirring for 2 h. The reaction mixture was then quenched with water and extracted with 1 M NaOH solution (2 × 100 mL). The water phases were combined, washed with CH_2_Cl_2_ (50 mL) and acidified with 6 M HCl until the solution tested acidic by litmus paper. The precipitate was filtrated, washed with water and CH_2_Cl_2_. The title product was obtained after drying under high vacuum as white powder, 640 mg, 3.14 mmol, 52%.

[α]_D_^20^ = −134 (c = 0.00203, EtOH). ^1^H-NMR (400 MHz, DMSO-*d*_6_) δ/ppm = 12.4 (s, 2H, 2 × COOH), 7.04 (d, *J* = 2.0 Hz, 2H, 2 × C_Ar_H), 6.78 (dd, *J* = 7.8, 1.9 Hz, 2H, 2 × C_Ar_H), 6.60 (d, *J* = 7.8 Hz, 2H, 2 × C_Ar_H), 4.04–3.88 (m, 2H, 2 × C_Ar_H), 3.15 (dd, *J* = 12.5, 9.8 Hz, 2H, 2 × C_Ar_H), 2.98 (ddd, *J* = 12.5, 9.6, 7.3 Hz, 2H, C_Ar_H), 2.81 (ddd, *J* = 12.3, 9.8, 7.3 Hz, 2H, 2 × C_Ar_H). ^13^C-NMR (101 MHz, DMSO-*d*_6_) δ/ppm = 167.7 (C_q_, 2 × COOH), 141.9 (C_q_, 2 × C_Ar_), 139.7 (C_q_, 2 × C_Ar_), 136.1 (+, CH, 2 × C_Ar_), 135.9 (+, CH, 2 × C_Ar_), 133.3 (+, CH, 2 × C_Ar_), 130.7 (C_q_, 2 × C_Ar_-COOH), 35.3 (−, CH_2_, 2 × C^PC^), 33.7 (−, CH_2_, 2 × C^PC^). IR (ATR): $\tilde{v}$/cm^–1^ = 2925 (w), 1674 (w), 1592 (w), 1556 (w), 1489 (vw), 1422 (w), 1300 (w), 1273 (w), 1203 (w), 1074 (w), 909 (w), 850 (vw), 797 (vw), 759 (vw), 717 (vw), 664 (w), 631 (w), 555 (vw), 518 (w), 426 (vw). MS (70 eV, EI) *m*/*z (%)*: 296 (27) [M]^+^, 278 (100) [MH_2_O]^+^, 148 (83) [MC_9_H_8_O_2_]^+^. HRMS (EI, C_18_H_16_O_4_) calc. 296.1049, found 296.1049. The analytical data match those reported in the literature[22].

*(R_p_,S)-4,12-Bis(4′-isopropyloxazolin-2′yl)[2.2]paracyclophane (R_p_,S)-**4a***

Thionyl chloride (1.0 mL) was added to (*R*_p_,*S*)-4,12-dicarboxy[2.2]paracyclophane (250 mg, 0.840 mmol, 1.00 equiv.) and the resulting mixture was stirred at 100 °C for 90 min. After cooling to room temperature, the excess thionyl chloride was removed under vacuum, the final traces were washed with toluene (2 × 2 mL) and removed under vacuum. The resulting crude acetyl chloride was dissolved in CH_2_Cl_2_ (5 mL) and cooled to 0 °C. A solution of *S*-Valinol (0.350 g, 3.36 mmol, 4.00 equiv.) and Et_3_N (0.54 mL, 0.42 g, 4.20 mmol, 5.00 equiv.) in CH_2_Cl_2_ (1.0 mL) was added, the reaction mixture was allowed to warm to room temperature and stirred for 24 h. 10 mL of CH_2_Cl_2_ was then added and the solution was washed with aq. NaHCO_3_ solution (3.5% *w*/*v*, 2 × 10 mL) and brine (20 mL). The organic phase was dried over MgSO_4_, filtered, concentrated, and dried under vacuum to give the crude amide as light brown solid.

The crude amide was dissolved in CH_3_CN (5.0 mL), PPh_3_ (0.66 g, 2.52 mmol, 3.00 equiv.), CCl_4_ (0.770 mL, 1.23 g, 7.98 mmol, 9.50 equiv.) and Et_3_N (0.970 mL, 0.760 g, 7.56 mmol, 9.00 equiv.) were added subsequently. After stirring at room temperature overnight, the solvent was removed under reduced pressure, the resulting mixture was dissolved in CH_2_Cl_2_ and washed with H_2_O (2 × 10 mL), the combined organic phase was washed with brine, dried over Na_2_SO_4_, filtrated and concentrated under vacuum. The crude was purified via column chromatography (*c*-Hex/EtOAc = 9:1) to give the title product as a pale yellow solid, 0.150 g, 0.350 mmol, 42%.

*R_f_* = 0.34 (*c*-Hex/EtOAc = 9:1). ^1^H-NMR (400 MHz, CDCl_3_) δ/ppm = 7.09 (d, *J* = 1.9 Hz, 2H, 2 × C_Ar_H), 6.62 (dd, *J* = 7.9, 1.9 Hz, 2H, 2 × C_Ar_H), 6.54 (d, *J* = 7.8 Hz, 2H, 2 × C_Ar_H), 4.37 (ddd, *J* = 11.2, 9.5, 2.0 Hz, 2H, 2 × CH_PC_), 4.30 (dd, *J* = 5.8, 2.2 Hz, 2H, 2 × CH^5^^′^), 4.04 (dd, *J* = 8.6, 6.7 Hz, 2H, 2 × CH^4^^′^), 4.05–3.92 (m, 2H, 2 × CH^5^^′^), 3.24–3.16 (m, 2H, 2 × CH_PC_), 3.16–3.07 (m, 2H, 2 × CH_PC_), 2.82 (ddd, *J* = 12.6, 10.0, 7.1 Hz, 2H, 2 × CH_PC_), 1.95 (hept, *J* = 6.7 Hz, 2H, 2 × CH^6^^′^), 1.20 (d, *J* = 6.7 Hz, 6H, CH^7^^′^), 1.06 (d, *J* = 6.7 Hz, 6H, CH^7^^′^). ^13^C-NMR (101 MHz, CDCl_3_) δ/ppm = 162.9 (C_q_, 2 × C^2^^′^), 141.0 (C_q_, 2 × *C*_Ar_*)*, 140.1 (C_q_, 2 × *C*_Ar_*)*, 135.8 (+, CH, 2 × *C*_Ar_*)*, 134.8 (+, CH, 2 × *C*_Ar_*)*, 132.3 (+, CH, 2 × *C*_Ar_*)*, 128.2 (C_q_, 2 × *C*_Ar_*)*, 73.8 (+, CH, 2 × *C*^4^^′^), 69.3 (−, CH_2_, 2 × *C*^5^^′^), 35.8 (−, CH_2_, 2 × *C*^PC^), 33.6 (+, CH, 2 × *C*^6^^′^), 33.6 (−, CH_2_, 2 × *C*^PC^), 19.7 (+, CH_3_, 2 × *C*^7^^′^), 19.2 (+, CH_3_, 2 × *C*^7^^′^). IR (ATR): $\tilde{v}$/cm^–1^ = 2955 (w), 1637 (m), 1590 (w), 1492 (w), 1468 (w), 1429 (w), 1384 (w), 1346 (w), 1303 (w), 1275 (w), 1258 (w), 1191 (w), 1172 (w), 1137 (w), 1115 (w), 1053 (m), 1026 (w), 984 (m), 933 (w), 907 (m), 889 (w), 822 (w), 749 (w), 694 (w), 674 (w), 643 (w), 514 (w), 482 (vw), 389 (vw). MS (FAB, 3-NBA), *m*/*z* (%): 431 (100) [M + H]^+^, 500/488 (9/9) [C_14_H_17_NO_2_ + H]^+^. HRMS (FAB, C_28_H_35_O_2_N_2,_ [M + H]^+^): calc. 431.2699, found 431.2701.

*(R_p_,S)-4,12-Bis(4′-tertbutyloxazolin-2′yl)[2.2]paracyclophane (R_p_,S)-**4b***

Thionyl chloride (2.0 mL) was added to (*R*_p_)-4,12-dicarboxy[2.2]paracyclophane (0.150 g, 0.510 mmol, 1.00 equiv.), after stirring at room temperature for 10 min, the mixture was heated to 100 °C and stirred at this temperature for 90 min. The excess thionyl chloride was removed by evaporation, the final traces were washed with toluene (2 × 2 mL). After drying under vacuum, the resulting crude acid chloride was dissolved in abs. CH_2_Cl_2_ (5 mL) and cooled to 0 °C. A solution of (*S*)-(+)-*tert*-leucinol (0.229 g, 2.04 mmol, 4.00 equiv.) and abs. Et_3_N (0.260 g, 0.360 mL, 2.55 mmol, 5.00 equiv.) in CH_2_Cl_2_ (1.0 mL) was added and the reaction mixture allowed to warm to room temperature and stirred for 24 h. Water was then added (10 mL) and extracted with CH_2_Cl_2_ (3 × 10 mL), the combined organic phase was washed with sat. NaHCO_3_ solution and brine (20 mL). The organic phase was dried over MgSO_4_, filtrated, concentrated, and dried under vacuum. The crude was purified via column chromatography (CH_2_Cl_2_/MeOH = 98:2 → 95:5) to give the intermediate amide. To a solution of this amide (152 mg, 0.307 mmol, 1.00 equiv.) and PPh_3_ (282 mg, 1.08 mmol, 3.50 equiv.) in abs. CH_3_CN (8.00 mL) was added triethyl amine (0.385 mL, 280 mg, 2.76 mmol, 9.00 equiv.) and CCl_4_ (0.281 mL, 449 mg, 2.92 mmol, 9.50 equiv.) under argon atmosphere. After stirring at room temperature overnight, the solvent was removed under vacuum, the resulting crude was dissolved in CH_2_Cl_2_ and washed with brine, the organic phase was dried over Na_2_SO_4_, filtrated and concentrated under vacuum. The resulting mixture was purified via column chromatography (*c*-Hex/EtOAc = 9:1) to give the title product as colorless solid, 104 mg, 0.227 mmol, 44% over two steps.

*R_f_* = 0.36 (*c*-Hex/EtOAc = 9:1). ^1^H-NMR (400 MHz, CDCl_3_) δ/ppm = 7.12 (d, *J* = 1.9 Hz, 2H, 2 × C_Ar_H), 6.64 (dd, *J* = 7.8, 1.9 Hz, 2H, 2 × C_Ar_H), 6.55 (d, *J* = 7.8 Hz, 2H, 2 × C_Ar_H), 4.33–4.24 (m, 2H, 2 × CH^5^^′^), 4.20 (td, *J* = 8.8, 3.7 Hz, 2H, 2 × CH^4^^′^), 4.17–4.08 (m, 4H, 2 × CH^5^^′^ + 2 × CH_PC_), 3.21–3.03 (m, 4H, 4 × CH_PC_), 2.86–2.68 (m, 2H, 2 × CH_PC_), 0.99 (s, 18H, CH^7^^′^). ^13^C-NMR (101 MHz, CDCl_3_) δ/ppm = 162.9 (C_q_, 2 × C^2^^′^), 141.0 (C_q_, 2 × C_Ar_), 140.2 (C_q_, 2 × C_Ar_), 135.6 (+, CH, 2 × C_Ar_), 134.7 (+, CH, 2 × C_Ar_), 132.2 (+, CH, 2 × C_Ar_), 128.0 (C_q,_ 2 × C_Ar_), 74.2 (+, CH, 2 × C^4^^′^), 67.7 (−, CH_2_, 2 × C^5^^′^), 36.2 (−, CH_2,_ 2 × C^PC^), 34.1 (−, CH_2_, 2 × C^PC^), 34.0 (C_q_, 2 × C^6^^′^), 26.1 (+, CH_3_, 6 × C^7^^′^). IR (ATR): $\tilde{v}$/cm^−1^ = 2951 (w), 2866 (w), 1638 (m), 1590 (w), 1477 (w), 1392 (w), 1350 (w), 1333 (w), 1303 (w), 1257 (w), 1191 (w), 1172 (w), 1113 (w), 1067 (w), 1047 (w), 1024 (w), 979 (m), 930 (w), 906 (w), 819 (w), 791 (w), 719 (w), 679 (w), 632 (w), 544 (vw), 513 (w). MS (FAB, 3-NBA), *m*/*z* (%): 459 (82) [M + H]^+^, 230 (75) [C_15_H_19_NO + H]^+^. HRMS (FAB, C_30_H_39_O_2_N_2,_ [M + H]^+^): calc. 459.3012, found 459.3011.

*(R_p_,S)-4,12-Bis(1′-phenyloxazolin-2′yl)[2.2]paracyclophane (R_p_,S)-**4c***

Thionyl chloride (2.0 mL) was added to (*R*_p_)-4,12-dicarboxy[2.2]paracyclophane (0.150 g, 0.510 mmol, 1.00 equiv.), after stirring at room temperature for 10 min, the mixture was heated to 100 °C and stirred under this temperature for 90 min. The excess thionyl chloride was removed by evaporation and the final traces were washed with toluene (2 × 2 mL). After drying under vacuum, the resulting crude acid chloride was dissolved in abs. CH_2_Cl_2_ (5 mL) and cooled to 0 °C. A solution of (*S*)-(+)-phenylglycinol (0.280 g, 2.04 mmol, 4.00 equiv.) and abs. Et_3_N (0.360 mL, 0.260 g, 2.55 mmol, 5.00 equiv.) in CH_2_Cl_2_ (1 mL) was added and the reaction mixture allowed to warm to room temperature and stirred for 24 h. Water (10 mL) was then added, the water phase was extracted with CH_2_Cl_2_ (3 × 10 mL) and the combined organic phase was washed with sat. NaHCO_3_ solution and brine (20 mL). The organic phase was dried over MgSO_4_, filtered, concentrated, and dried in vacuum. The crude was purified via column chromatography (CH_2_Cl_2_/MeOH = 98:2 → 95:5) to give the intermediate amide. To a solution of this amide (200 mg, 0.374 mmol, 1.00 equiv.) and PPh_3_ (344 mg, 1.31 mmol, 3.50 equiv.) in abs. 10 mL of CH_3_CN was added Et_3_N (0.469 mL, 341 mg, 3.37 mmol, 9.00 equiv.) and CCl_4_ (0.343 mL, 547 mg, 3.55 mmol, 9.50 equiv.) under argon atmosphere. After stirring at room temperature overnight, the solvent was removed under vacuum, the resulting crude was dissolved in CH_2_Cl_2_ and washed with brine. The organic phase was dried over Na_2_SO_4_, filtrated and concentrated under vacuum, the resulting mixture was purified via column chromatography (*c*-Hex/EtOAc = 9:1) to give the title product as colorless solid, 177 mg, 0.355 mmol, 95%.

*R**_f_*** = 0.14 (*c*-Hex/EtOAc = 9:1). ^1^H-NMR (500 MHz, CDCl_3_) δ/ppm = 7.40–7.29 (m, 12H, CH^7 + 8′ + 9^ + 2 × C_Ar_H), 6.70 (dd, *J* = 7.9, 1.9 Hz, 2H, 2 × C_Ar_H), 6.61 (d, *J* = 7.9 Hz, 2H, 2 × C_Ar_H), 5.47 (dd, *J* = 10.1, 8.2 Hz, 2H, 2 × CH^5^^′^), 4.65 (dd, J = 10.1, 8.2 Hz, 2H, 2 × CH^5^^′^), 4.44–4.25 (m, 2H, 2 × CH_PC_), 4.13 (t, J = 8.2 Hz, 2H, 2 × CH^4^^′^), 3.22–3.14 (m, 4H, 4 × CH_PC_), 2.89–2.79 (m, 2H, 2 × CH_PC_). ^13^C-NMR (126 MHz, CDCl_3_) δ/ppm = 164.6 (C_q_, 2 × C^2^^′^), 143.0 (C_q_, 2 × C^6^^′^), 141.4 (C_q_, 2 × C_Ar_), 140.3 (C_q_, 2 × C_Ar_), 135.9 (+, CH, 2 × C_Ar_), 135.1 (+, CH, 2 × C_Ar_), 132.8 (+, CH, 2 × C_Ar_), 128.8 (+, CH, 4 × C^8^^′^), 128.5 (C_q_, CH, 2 × C_Ar_), 127.5 (+, CH, 2 × C^9^^′^), 126.9 (+, CH, 4 × C^7^^′^), 73.9 (+, CH, 2 × C^4^^′^), 70.7 (−, CH_2_, 2 × C^5^^′^), 36.4 (−, CH_2_, 2 × C^PC^), 34.2 (−, CH_2_, 2 × C^PC^). IR (ATR): $\tilde{v}$/cm^–1^ = 2922 (w), 1630 (m), 1589 (w), 1493 (w), 1448 (w), 1349 (w), 1296 (w), 1274 (w), 1245 (w), 1191 (w), 1172 (w), 1136 (vw), 1116 (vw), 1050 (w), 986 (w), 961 (w), 927 (w), 902 (w), 887 (w), 823 (w), 750 (w), 697 (w), 639 (w), 523 (w), 388 (vw). MS (FAB, 3-NBA), *m*/*z* (%): 499 (100) [M + H]^+^, 250 (34) [C_17_H_15_NO + H]^+^. HRMS (FAB, C_34_H_31_O_2_N_2,_ [M + H]^+^): calc 499.2386, found 499.2386.

*Methyl 2-Phenyl-2-(phenylamino)acetate (**7a**)*

General procedure (GP) was followed by adding phenyl-2-diazopropionate (17.6 mg, 1.00 mmol, 1.00 equiv.) and aniline (11.2 mg, 1.20 mmol, 1.20 equiv.) to a suspension of in situ generated Cu-(*R_p_,S*)-4a catalyst. The product 7a was obtained via flash chromatography (*c*-Hex/EtOAc = 5:1) as colorless solid, 23.6 mg, 0.98 mmol, 98%.

^1^H-NMR (300 MHz, CDCl_3_) δ/ppm = 7.42 (d, *J* = 7.7 Hz, 2H, *2 ×* C_Ar_H), 7.27 (qd, *J* = 7.5, 6.4, 2.6 Hz, 3H, 3 × C_Ar_H), 7.04 (t, *J* = 7.9 Hz, 2H, 2 × C_Ar_H), 6.62 (t, *J* = 7.3 Hz, 1H, C_Ar_H), 6.48 (d, *J* = 7.7 Hz, 2H, 2 × C_Ar_H), 5.01 (d, *J* = 5.9 Hz, 1H, CHN), 4.88 (s, 1H, NH), 3.65 (s, 3H, OCH_3_).

The analytical data matches the data reported in the literature [23].

*Methyl 2-phenyl-2-(phenylamino)acetate (**7b**)*

GP was followed by adding phenyl-2-diazopropionate (17.6 mg, 1.00 mmol,1.00 equiv.) and aniline (11.2 mg, 1.20 mmol, 1.20 equiv.) to a suspension of in situ generated Cu-(*R_p_,S*)-**4a** catalyst. The product **7b** was obtained via flash chromatography (*c*-Hex/EtOAc = 5:1) as colorless solid, 23.6 mg, 0.98 mmol, 98%.

^1^H-NMR (300 MHz, CDCl_3_) δ/ppm = 7.42 (d, *J* = 7.7 Hz, 2H, *2 ×* C_Ar_H), 7.27 (qd, *J* = 7.5, 6.4, 2.6 Hz, 3H, 3 × C_Ar_H), 7.04 (t, *J* = 7.9 Hz, 2H, 2 × C_Ar_H), 6.62 (t, *J* = 7.3 Hz, 1H, C_Ar_H), 6.48 (d, *J* = 7.7 Hz, 2H, 2 × C_Ar_H), 5.01 (d, *J* = 5.9 Hz, 1H, CHN), 4.88 (s, 1H, NH), 3.65 (s, 3H, OCH_3_).

The analytical data matches the data reported in the literature[23].

*General Procedure (GP): Copper-Catalyzed N–H Insertion*

Cu(MeCN)_4_PF_6_ (5 mol%), ligand (6 mol%) and NaBArF (6 mol%) were added into an oven-dried screw vial, evacuated, and backfilled with argon three times. After CH_2_Cl_2_ (1 mL) was injected into the vial, the solution was stirred at 40 °C under argon atmosphere overnight. A solution of *α*-diazopropionates (1.00 equiv.) and aniline (1.20 equiv.) in CH_2_Cl_2_ (1 mL) was added dropwise, the mixture was stirred at room temperature for 2 h. The resulting mixture was dried under vacuum and purified via column chromatography (*c*-Hex/EtOAc = 8:1 or pentane/Et_2_O = 5:1) to give the products **7a**–**j**.

*Benzyl phenylalaninate (**7c**)*

GP was followed by adding benzyl 2-diazopropanoate (19.0 mg, 1.00 mmol, 1.00 equiv.) and aniline (11.2 mg, 1.20 mmol, 1.20 equiv.) to a suspension of in situ generated Cu-(*R*_p_,*S*)-**4a** catalyst. The product was obtained as a light-yellow solid (*c*-Hex/EtOAc = 4:1), 23.9 mg, 0.94 mmol, 94%.

*R_f_* = 0.33 (*c*-Hex/EtOAc = 5:1). ^1^H-NMR (300 MHz, CDCl_3_) δ/ppm = 7.31–7.16 (m, 5H, CH_2_Ph), 7.15–7.02 (m, 2H, 2 × C_Ar_H*)*, 6.73 (tt, *J* = 7.3, 1.1 Hz, 1H, C_Ar_H), 6.63 (dd, *J* = 8.6, 1.2 Hz, 2H, 2 × C_Ar_H), 5.07 (s, 2H, CH_2_Ph), 4.14 (q, *J* = 7.0 Hz, 1H, CHN), 3.98 (s, 1H, NH), 1.42 (d, *J* = 7.0 Hz, 3H, CHCH_3_).

The analytical data matches the data reported in the literature [24].

*Benzyl (2-methoxyphenyl)alaninate (**7d**)*

GP was followed by adding benzyl 2-diazopropanoate (19.0 mg, 1.00 mmol, 1.00 equiv.) and *o*-anisidine (14.8 mg, 1.20 mmol, 1.20 equiv.) to a suspension of in situ generated Cu-(*R*_p_,*S*)-**4a** catalyst. The product was obtained via column chromatography (*c*-Hex/EtOAc = 5:1) as a light-yellow solid, 23.3 mg, 0.82 mmol, 82%.

^1^H-NMR (300 MHz, CDCl_3_) δ/ppm = 7.40–7.06 (m, 5H, CH_2_Ph), 6.78–6.66 (m, 2H, 2 × C_Ar_H), 6.62 (ddd, *J* = 8.2, 7.2, 1.6 Hz, 1H, C_Ar_H), 6.44 (dd, *J* = 7.6, 1.6 Hz, 1H, C_Ar_H), 5.07 (s, 2H, CH_2_Ph), 4.64 (s, 1H, NH), 4.12 (q, *J* = 7.0 Hz, 1H, CHN), 3.76 (s, 3H, OCH_3_), 1.44 (d, *J* = 6.9 Hz, 3H, CHCH_3_). ^13^C-NMR (75 MHz, CDCl_3_) δ/ppm = 174.5 (C_q_, CO_2_Bn), 147.2 (C_q_, C_Ar_), 136.6 (C_q_, C_Ar_), 135.8 (C_q_, C_Ar_), 128.6 (+, CH, 2 × C_Ar_), 128.3 (+, CH, C_Ar_), 128.2 (+, CH, 2 × C_Ar_), 121.3 (+, CH, C_Ar_), 117.7 (+, CH, C_Ar_), 110.6 (+, CH, C_Ar_), 109.9 (+, CH, C_Ar_), 66.8 (−, CH_2_, CH_2_Ph), 55.5(+, CH_3_, OCH_3_), 52.0(+, CH, CHN), 18.9 (+, CH_3_).

The analytical data matches the data reported in the literature [24].

*Benzyl (3-Methoxyphenyl)alaninate (**7e**)*

GP was followed by adding benzyl 2-diazopropanoate (19.0 mg, 1.00 mmol, 1.00 equiv.) and *m-*anisidine (14.8 mg, 1.20 mmol, 1.20 equiv.) to a suspension of in situ generated Cu-(*R*_p_,*S*)-**4a** catalyst. The product was obtained via column chromatography (*c*-Hex/EtOAc = 5:1) as light-yellow solid, 15.1 mg, 53%.

^1^H-NMR (300 MHz, CDCl_3_) δ/ppm = 7.39–7.27 (m, 5H, CH_2_*Ph*), 7.07 (t, *J* = 8.1 Hz, 1H, C_Ar_H), 6.32 (dd, *J* = 8.2, 2.3 Hz, 1H, C_Ar_H), 6.25–6.19 (m, 1H, C_Ar_H), 6.16 (t, *J* = 2.3 Hz, 1H, C_Ar_H), 5.16 (s, 2H, CH_2_Ph), 4.19 (q, *J* = 6.9 Hz, 1H, CHN), 3.74 (s, 1H, NH), 1.48 (d, *J* = 6.9 Hz, 3H, CHCH_3_). ^13^C-NMR (75 MHz, CDCl_3_) δ/ppm = 174.3 (C_q_, *C*O_2_Bn), 160.8 (C_q_, C_Ar_), 147.9 (C_q_, C_Ar_), 135.5 (C_q_, C_Ar_), 130.1 (+, CH, C_Ar_), 128.5 (+, CH, 2 × C_Ar_), 128.3 (+, CH, C_Ar_), 128.1 (+, CH, 2 × C_Ar_), 106.3 (+, CH, C_Ar_), 103.7 (+, CH, C_Ar_), 99.5 (+, CH, C_Ar_), 66.8 (−, CH_2_, CH_2_Ph), 55.0 (+, CH_3_, OCH_3_), 52.0 (+, CH, CHN), 18.8 (+, CH_3_).

*Benzyl (4-methoxyphenyl)alaninate (**7f**)*

GP was followed by adding benzyl 2-diazopropanoate (19.0 mg, 1.00 mmol, 1.00 equiv.) and *p-*anisidine (14.8 mg, 1.20 mmol, 1.20 equiv.) to a suspension of in situ generated Cu-(*R*_p_,*S*)-**4a** catalyst. The product was obtained via column chromatography (*c*-Hex/EtOAc = 4:1) as light-yellow solid, 20.0 mg, 70%.

^1^H-NMR (300 MHz, CDCl_3_) δ/ppm = 7.38–7.03 (m, 5H, CH_2_Ph), 6.72–6.61 (m, 2H, 2 × C_Ar_H), 6.51 (d, *J* = 9.0 Hz, 2H, 2 × C_Ar_H), 5.06 (s, 2H, CH_2_Ph), 4.04 (q, *J* = 6.9 Hz, 1H, CHN), 3.66 (s, 3H, CH_3_). ^13^C-NMR (75 MHz, CDCl_3_) δ/ppm = 175.3 (C_q_, CO_2_Bn), 153.4 (C_q_, C_Ar_), 141.2 89 (C_q_, C_Ar_), 136.1 (C_q_, C_Ar_), 129.1 (+, CH, 2 × C_Ar_), 128.8 (+, CH, C_Ar_), 128.6 (+, CH, 2 × C_Ar_), 115.7 (+, CH, 2 × C_Ar_), 115.4 (+, CH, 2 × C_Ar_), 67.2 (−, CH_2_, CH_2_Ph), 56.2 (+, CH_3_ OCH_3_,), 53.8 (+, CH, CHN), 19.5 (+, *C*H_3_).

The analytical data matches the data reported in the literature[25].

*Benzyl o-tolylalaninate (**7g**)*

GP was followed by adding benzyl 2-diazopropanoate (19.0 mg, 1.00 mmol, 1.00 equiv.) and *o*-toluidine (12.9 mg, 1.20 mmol, 1.20 equiv.) to a suspension of in situ generated Cu-(*R*_p_,*S*)-**4a** catalyst. The product was obtained via column chromatography (*c*-Hex/EtOAc = 5:1) as colorless solid, 18.3 mg, 0.68 mmol, 68%.

^1^H-NMR (300 MHz, CDCl_3_) δ/ppm = 7.44–7.27 (m, 5H, CH_2_Ph), 7.15–7.05 (m, 2H, Ph), 6.72 (td, *J* = 7.4, 1.2 Hz, 1H, Ph), 6.55 (dd, *J* = 8.4, 1.2 Hz, 1H, Ph), 5.19 (s, 2H, CH_2_Ph), 4.27 (q, *J* = 6.9 Hz, 1H, CHCH_3_), 4.08 (s, 1H, NH), 2.21 (s, 3H, CH_3_), 1.55 (d, *J* = 6.9 Hz, 3H, CHCH_3_). ^13^C-NMR (75 MHz, CDCl_3_) δ/ppm = 174.7 (C_q_, *C*O_2_Bn), 144.7 (C_q_, C_Ar_), 135.7 (C_q_, C_Ar_), 130.5 (C_q_, C_Ar_), 128.7 (+, CH, 2 × C_Ar_), 128.4 (+, CH, C_Ar_), 128.2 (+, CH, 2 × C_Ar_), 127.2 (+, CH, C_Ar_), 122.8 (+, CH, C_Ar_), 118.0 (+, CH, C_Ar_), 110.5 (+, CH, C_Ar_), 66.9 (−, CH_2_, CH_2_Ph), 52.1 (+, CH, CHN), 19.2 (+, CH_3_), 17.5 (+, CH_3_).

*Benzyl p-tolylalaninate (**7h**)*

GP was followed by adding benzyl 2-diazopropanoate (19.0 mg, 1.00 mmol, 1.00 equiv.) and *p*-toluidine (12.9 mg, 1.20 mmol, 1.20 equiv.) to a suspension of in situ generated Cu-(*S*_p_,*S*)/(*R*_p_,*S*)-**4a** catalyst. The product was obtained via column chromatography (*c*-Hex/EtOAc = 4:1) as light-yellow solid, 18.8 mg, 0.70 mmol, 70%.

R_f_ = 0.31 (*c*-Hex/EtOAc = 5:1). ^1^H-NMR (300 MHz, CDCl_3_) δ/ppm = 7.34–7.14 (m, 5H, CH_2_*Ph*), 6.89 (d, *J*=8.1 Hz, 2H, 2 × C_Ar_H), 6.45 (d, *J* = 8.4 Hz, 2H, 2 × C_Ar_H), 5.06 (s, 2H, CH_2_Ph), 4.09 (q, *J* = 7.0 Hz, 1H, CHN), 3.93 (s, 1H, NH), 2.16 (s, 3H, CH_3_), 1.39 (d, *J* = 6.9 Hz, 3H, CHCH_3_). ^13^C-NMR (75 MHz, CDCl_3_) δ/ppm = 174.7 (C_q,_ CO_2_Bn), 144.4 (C_q_, C_Ar_), 135.7 (C_q_, C_Ar_), 129.9 (C_q_, C_Ar_), 128.6 (+, CH, 2 × C_Ar_H), 128.4 (+, CH, C_Ar_H), 128.2 (+, CH, 2 × C_Ar_H), 127.8 (+, CH, C_Ar_H), 113.9 (+, CH, C_Ar_H), 66.8 (−, CH_2_, CH_2_Ph), 52.6 (+, CH, CHN), 20.5 (+, CH_3_), 19.0 (+, CH_3_).

*Phenyl phenylalaninate (**7i**)*

GP was followed by adding methyl 2-diazo-2-phenylacetate (17.6 mg, 1.00 mmol, 1.00 equiv.) and aniline (11.2 mg, 1.20 mmol, 1.20 equiv.) to a suspension of in situ generated Cu-(*R*p,*S*)-**4a** catalyst. The product was obtained via column chromatography (*c*-Hex/EtOAc = 5:1) as light-yellow liquid, 16.3 mg, 0.68 mmol, 68%.

^1^H-NMR (300 MHz, CDCl_3_) δ/ppm = 7.29 (td, *J* = 7.4, 6.8, 1.3 Hz, 2H, 2 × C_Ar_H), 7.20–7.03 (m, 3H, 3 × C_Ar_H), 6.98–6.87 (m, 2H, 2 × C_Ar_H), 6.72 (tt, *J* = 7.3, 1.1 Hz, 1H, C_Ar_H), 6.64 (dt, *J* = 7.7, 1.1 Hz, 2H, 2 × C_Ar_H), 4.32 (q, *J* = 7.0 Hz, 1H, CHN), 4.12 (s, 1H, NH), 1.58 (d, *J* = 6.9 Hz, 3H, CHCH_3_).

*tert-Butyl phenylalaninate (**7j**)*

GP was followed by adding *tert*-Butyl 2-diazopropanoate (15.6 mg, 1.00 mmol, 1.00 equiv.) and aniline (11.2 mg, 1.20 mmol, 1.20 equiv.) to a suspension of in situ generated Cu-(*R_p_,S*)-**4a** catalyst. The product was obtained via flash chromatography (*c*-Hex/EtOAc = 8:1) as a light-yellow liquid, 16.4 mg, 0.74 mmol, 74%.

^1^H-NMR (300 MHz, CDCl_3_) δ/ppm = 7.21–7.11 (m, 3H, C_Ar_H), 6.73 (t, *J* = 7.3 Hz, 1H, C_Ar_H), 6.61 (d, *J* = 7.7 Hz, 2H, C_Ar_H), 4.02 (q, *J* = 6.9 Hz, 1H, CHN), 1.44 (s, 9H, C(CH_3_)_3_), 1.43 (d, *J* = 6.9 Hz, 3H). – ^13^C-NMR (75 MHz, CDCl_3_) δ/ppm = 174.3 (C_q_, *C*O_2_*t*Bu), 147.3 (C_q_, C_Ar_*)*, 129.8 (+, CH, 2 × C_Ar_*)*, 118.6 (+, CH, C_Ar_*)*, 114.0 (+, CH, 2 × C_Ar_*)*, 82.0 (C_q_, C(CH_3_)_3_), 53.1 (+, CH, CHNH), 28.5 (+, CH_3_, 3 × CH_3_), 19.4 (+, CH_3_, CHCH_3_).

The analytical data matches the data reported in the literature [26].

*Methyl 2-phenyl-2-(phenylamino)acetate (**14**)*

GP was followed by adding phenyl-2-diazopropionate (17.6 mg, 1.00 mmol,1.00 equiv.) and aniline (11.2 mg, 1.20 mmol, 1.20 equiv.) to a suspension of in situ generated Cu-(*R_p_,S*)-4a catalyst. The product 7b was obtained via flash chromatography (*c*-Hex/EtOAc = 5:1) as colorless solid, 23.6 mg, 0.98 mmol, 98%.

^1^H-NMR (300 MHz, CDCl_3_) δ/ppm = 7.42 (d, *J* = 7.7 Hz, 2H, *2 ×* C_Ar_H), 7.27 (qd, *J* = 7.5, 6.4, 2.6 Hz, 3H, 3 × C_Ar_H), 7.04 (t, *J* = 7.9 Hz, 2H, 2 × C_Ar_H), 6.62 (t, *J* = 7.3 Hz, 1H, C_Ar_H), 6.48 (d, *J* = 7.7 Hz, 2H, 2 × C_Ar_H), 5.01 (d, *J* = 5.9 Hz, 1H, CHN), 4.88 (s, 1H, NH), 3.65 (s, 3H, OCH_3_).

The analytical data matches the data reported in the literature [23].

## 4. Conclusions

The successful N–H insertion of α-diazocarbonyls into anilines by copper catalysis with PCPBOX ligands have been demonstrated. In this work, we showed the synthesis and catalytic application of three different PCPBOX ligands. Their straight-forward synthesis renders them a very accessible ligand system. The N–H insertion into saturated anilines was demonstrated to afford moderate to excellent yields with a wide substrate scope. The more sterically demanding PCPBOX ligands showed very good yields in the N–H insertion with unsaturated anilines.

## Figures and Tables

**Table 1:**
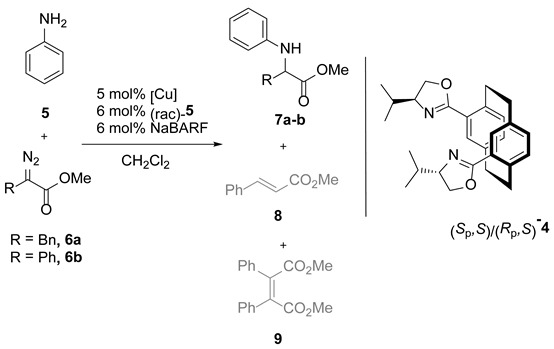
Optimization of the copper-catalyzed N–H insertion of diazocarbonyls **6a**–**b** into aniline **5**.

Entry	6	[Cu]	T [°C]	Yield [%] ^a^		
				7	8	9
1	**6a**	Cu(MeCN)_4_PF_6_	40	77	18	n/a
2		CuCl	40	–	88	n/a
3		[CuOTf]_2_·Tol	40	58	38	n/a
4		Cu(MeCN)_4_PF_6_	r.t.	93	5	n/a
5		[CuOTf]_2_·Tol	r.t.	61	22	n/a
6 ^b^		Cu(MeCN)_4_PF_6_	r.t.	13	84	n/a
7	**6b**	CuCl	r.t.	44	n/a	2
8		Cu(MeCN)_4_PF_6_	r.t.	98	n/a	2
9		[CuOTf]_2_·Tol	r.t.	64	n/a	28
10 ^b^		Cu(MeCN)_4_PF_6_	r.t.	40	n/a	–

^a^ Yields were determined by GC-MS. ^b^ no ligand **4**.

**Table 2:**
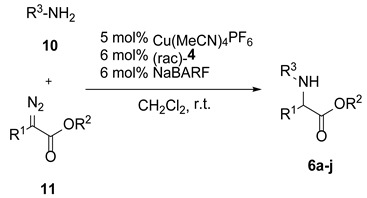
Optimization of copper-catalyzed N–H insertion of diazocarbonyl **11** into aniline **10**.

Entry	R^1^	R^2^	R^3^	Product	Yield [%] ^a^
1	Bn	Me	Ph	**7a**	77
2	Ph	Me	Ph	**7b**	98
3	Me	Bn	Ph	**7c**	94
4	Me	Bn	*o*-MeOPh	**7d**	82
5	Me	Bn	*m*-MeOPh	**7e**	53
6	Me	Bn	*p*-MeOPh	**7f**	70
7	Me	Bn	*o*-MePh	**7g**	68
8	Me	Bn	*p*-MePh	**7h**	70
9	Me	Ph	Ph	**7i**	68
10	Me	*t*Bu	Ph	**7j**	74
11	Me	Bn	*c*-C_6_H_11_	**7k**	–
12	Me	Ph	*c*-C_6_H_11_	**7l**	–

^a^ Isolated yields.

**Table 3:**
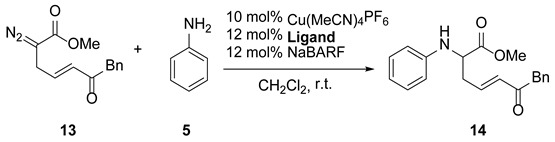
Catalyst screening for the N–H insertion of unsaturated α-diazocarbonyl **13**.

Entry	Ligand	Yield [%] ^a^
1	(*R_p_,S*)-**4a**	22
2	(*R_p_,S*)-**4a**	38
3	(*R_p_,S*)-**4b**	80
4	(*R_p_,S*)-**4c**	88

^a^ Isolated yields.

## Supplementary Materials

All NMR spectroscopic analysis and other characterization data are available online.
