# Supplementary material for: Planar Chiral [2.2]Paracyclophane-Based Bisoxazoline Ligands and Their Applications in Cu-Mediated N–H Insertion Reaction
Source: Molecules. 2019 Nov 14;24(22):4122. doi: 10.3390/molecules24224122 (PMC6891757; doi:10.3390/molecules24224122)
Supplement: Supplementary file 1 [file molecules-24-04122-s001.pdf]

molecules

Supplementary Materials

Planar Chiral [2.2]Paracyclophane-based BOX Ligands and Application in Cu-mediated N-H insertion reaction

Daniel M. Knoll,[1,†] Yuling Hu,[1,†] Zahid Hassan,[1] Martin Nieger,[2] Stefan Bräse*[1,3]

[1] Institute of Organic Chemistry (IOC), Karlsruhe Institute of Technology (KIT), Fritz-Haber-Weg 6, 76131 Karlsruhe, Germany.

[2] Department of Chemistry, University of Helsinki, P.O. Box 55 A.I. Virtasen aukio 1, 00014 University of Helsinki, Finland

[3] Institute of Toxicology and Genetics (ITG), Karlsruhe Institute of Technology (KIT), Hermann-von-Helmholtz-Platz 1, D-76344 Eggenstein-Leopoldshafen, Germany.

† Equal author contribution

* Correspondence: braese@kit.edu

Received: date; Accepted: date; Published: date

Abstract: New catalysts for important C–N bond formation are highly sought after. In this work, we demonstrate the synthesis, and viability of a new class of planar chiral [2.2]paracyclophane-based bisoxazoline (BOX) ligands for the copper catalyzed N-H insertion of α-diazocarbonyls into anilines. The reaction features a wide substrate scope, moderate to excellent yields and delivers the valuable products at ambient conditions.

Keywords: [2.2]paracyclophane ligand; N-H insertion; α-diazocarbonyls; planar chirality; copper catalysis.

Materials and Methods

4,16-Dibromo[2.2]paracyclophane (1)

A solution of Br_2 (5.50 mL, 17.0 g, 106 mmol, 2.20 equiv.) in CH_2Cl_2 (50 mL) was prepared. A suspension of iron powder (0.14 g, 2.4 mmol, 0.05 equiv.) in 6.25 mL of the Br_2/CH_2Cl_2 solution was diluted in 50 mL of CH_2Cl_2 and stirred at room temperature for 1 h. The solution was then brought to reflux for 2 h. CH_2Cl_2 (50 mL) and [2.2]paracyclophane (10.0 g, 48.0 mmol, 1.00 equiv.) were added to the mixture subsequently. After the remaining bromine solution was added dropwise over a period of 4 h, the mixture was stirred at room temperature for 3 d. Saturated $Na_2S_2O_3$ solution was added and the reaction mixture was stirred at room temperature until the bromine color disappeared. The organic phase was separated and filtrated, the precipitate was recrystallized from hot toluene to obtain the title product as an off-white solid, 5.40 g, 14.8 mmol, 31%.

^{1}H NMR (400 MHz, $CDCl_3$) δ/ppm = 7.14 (dd, *J* = 7.8, 1.8 Hz, 2H, 2 × $C_{Ar}H$), 6.51 (d, *J* = 1.8 Hz, 2H, 2 × $C_{Ar}H$), 6.44 (d, *J* = 7.8 Hz, 2H, 2 × $C_{Ar}H$) , 3.50 (ddd, *J* = 12.8, 10.3, 2.0 Hz, 2H, 2 × CH_{PC}), 3.16 (ddd, *J* = 12.1, 10.2, 4.6 Hz, 2H, 2 × CH_{PC}), 2.95 (ddd, *J* = 12.1, 11.4, 2.0 Hz, 2H, 2 × CH_{PC}), 2.85 (ddd, *J* = 13.0, 10.6, 4.6 Hz, 2H, 2 × CH_{PC}). – **^{13}C NMR** (101 MHz, $CDCl_3$) δ/ppm = 141.3 (C_q, 2 × C_{Ar}), 138.6 (C_q, 2 × C_{Ar}), 137.4 (+, CH, 2 × C_{Ar}), 134.2 (+, CH, 2 × C_{Ar}), 128.4 (+, CH, 2 × C_{Ar}), 126.8 (C_q, 2 × C_{Ar}-Br), 35.5 (–, 2 × CH_2), 32.9 51 (–, 2 × CH_2). – **IR** (ATR): $\tilde{\nu}$/cm^{-1} = 2932 (vw), 2849 (vw), 1895 (vw), 1583 (vw), 1532 (vw), 1474 (vw), 1449 (vw), 1432 (vw), 1390 (w), 1313 (vw), 1185 (vw), 1104 (vw), 1030 (w), 947 (vw), 899 (w), 839 (w), 855 (w), 830 (w), 706 (w), 669 (w), 647 (w), 522 (vw), 464 (w), 393 (vw). – **MS** (EI, 70 eV), m/z (%): 364/366/368 (3/6/3) $[M]^+$, 184/182 (18/18) $[M - C_8H_7Br]^+$, 104 (100) $[C_8H_8]^+$. – **HRMS** (EI, $C_{16}H_{14}{}^{79}Br_2$) calc. 363.9457, found 363.9455.

(*rac*)-4,12-Dibromo[2.2]paracyclophane (rac)-2

In a 10 mL microwave vessel was placed 4,16-dibromo[2.2]paracyclophane (500 mg, 1.37 mmol, 1.00 equiv.) and DMF (1.00 mL). The device was programmed to heat the mixture to 180 °C with a holding time set as 6 min. The maximum pressure for the system was set at 17.2 bar and the power was set at 300 W. After cooling to room temperature, the mixture was diluted with DMF (2 mL) and the precipitate was collected by filtration. The reaction was repeated under the same conditions until all the starting material (5.00 g, 13.7 mmol, 1.00 equiv.) reacted. The combined filtrate was poured into water (75 mL) and extracted with EtOAc (3 × 100 mL). The combined organic phase was washed with water and brine, dried over Na_2SO_4 and concentrated under reduced pressure to give the title product as a pale brown power, 3.50 g, 9.65 mmol, 70%.
$\boldsymbol{R_f}$ = 0.68 (*c*-Hex/EtOAc = 9:1). – ^{1}**H NMR** (400 MHz, $CDCl_3$) δ/ppm = 7.22 (d, *J* = 1.6 Hz, 2H, 2 × $C_{Ar}H$), 6.56 (d, *J* = 7.8 Hz, 2H, 2 × $C_{Ar}H$), 6.52 (dd, *J* = 7.9, 1.7 Hz, 2H, 2 × $C_{Ar}H$), 3.47 (ddd, *J* = 13.3, 9.6, 2.2 Hz, 2H, 2 × CH_{PC}), 3.10 (ddd, *J* = 13.0, 9.6, 6.8 Hz, 2H, 2 × CH_{PC}), 3.06–2.94 (m, 2H, 2 × CH_{PC}), 2.82 (ddd, *J* = 13.3, 10.1, 6.9 Hz, 2H, 2 × CH_{PC}). – ^{13}**C NMR** (101 MHz, $CDCl_3$) δ/ppm = 141.3 (C_q, 2 × C_{Ar}), 138.7 (C_q, 2 × C_{Ar}), 135.0 (+, CH, 2 × C_{Ar}), 132.7 (+, CH, 2 × C_{Ar}), 131.7 (+, CH, 2 × C_{Ar}), 126.7 (C_q, 2 × C_{Ar}-Br), 35.8 (–, 2 × CH_2), 32.5 (–, 2 × CH_2). – **IR** (ATR): $\tilde{\nu}$/cm^{-1} = 2923 (w), 2848 (w), 1583 (w), 1537 (w), 1474 (w), 1449 (w), 1431 (w), 1391 (m), 1272 (w), 1237 (w), 1201 (w), 1185 (w), 1030 (m), 902 (m), 858 (m), 785 (w), 705 (m), 644 (m), 475 (m). – **MS** (70 eV, EI) *m/z* (%): 368/366/364 (22/43/22) $[M]^+$, 288/286 (13/12) $[M+H–Br]^+$, 184/182 (80/100) $[M–C_8H_6Br]^+$, 104 (68) $[C_8H_8]^+$. – **HRMS** (EI, $C_{16}H_{14}{}^{79}Br_2$) calc. 363.9462, found 363.9461.

(R_P)-4,12-Dibromo[2.2]paracyclophane (R_p-2)

/ (S_P)-4,12-Dibromo[2.2]paracyclophane (S_p-2)

Separation of (*rac*)-4,12-dibromo[2.2]paracyclophane **75** *via* semi-preparative chiral HPLC:
Conditions:
Semi-preparative chiralpak® AZ-H column (20 × 250 nm, particle 5 μm), 100% CH_3CN, 25 mL/min, 25 °C, 254 nm UV detecter, 100 mg racemate per run. t_R (R_P) = 6.97 min, t_R (S_P) = 8.00 min. The spectra of the racemate as well as the enationpure (R_P-4,12-dibromo-[2.2]paracyclophane) **75** are shown in Figure 1.

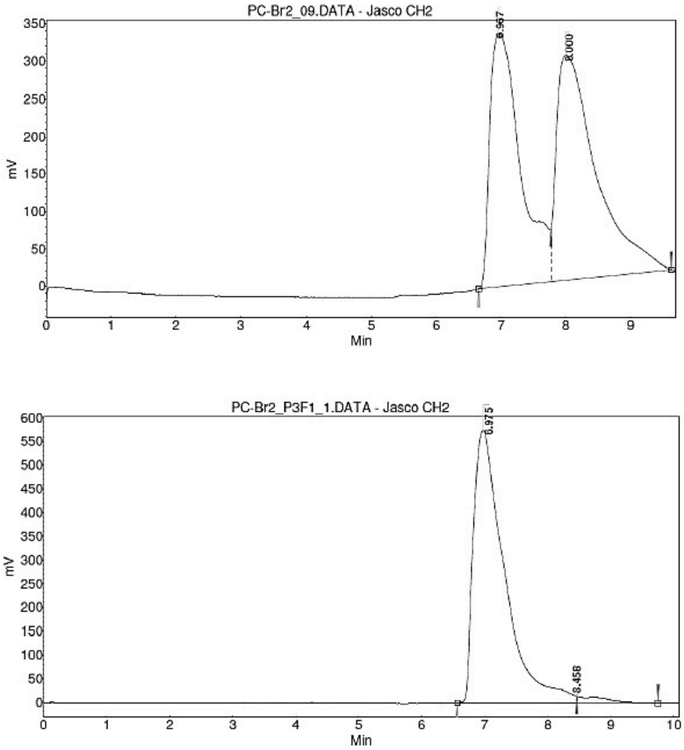

Figure 1. Separation of (*rac*)-4,12-dibromo[2.2]paracyclophane/(*rac*)-**75** *via* semi-preparative HPLC.

(*R*$_P$)-4,12-Dicarboxy[2.2]paracyclophane (*R*$_p$-3)

To a solution of (*R*$_P$)-4,12-dibromo[2.2]paracyclophane (1.50 g, 4.12 mmol, 1.00 equiv.) in abs. THF (50 mL) was added 9.71 mL of *t*-butyllithium (1.7 M in pentane, 15.4 mmol, 4.00 equiv.) dropwise at –78 °C. After stirring at – 78 °C for 3 h, CO_2 was bubbled through the solution *via* a long needle under stirring for 2 h. The reaction mixture was then quenched with water and extracted with 1 M NaOH solution (2 × 100 mL). The water phases were combined, washed with CH_2Cl_2 (50 mL) and acidified with 6 M HCl until the solution tested acidic by litmus paper. The precipitate was filtrated, washed with water and CH_2Cl_2. The title product was obtained after drying under high vacuum as white powder, 640 mg, 3.14 mmol, 52%.
[α]$_D^{20}$ = –134 (c = 0.00203, EtOH). – **^{1}H NMR** (400 MHz, DMSO-*d*$_6$) δ/ppm = 12.4 (s, 2H, 2 × COO*H*), 7.04 (d, *J* = 2.0 Hz, 2H, 2 × C_{Ar}H), 6.78 (dd, *J* = 7.8, 1.9 Hz, 2H, 2 × C_{Ar}H), 6.60 (d, *J* = 7.8 Hz, 2H, 2 × C_{Ar}H), 4.04–3.88 (m, 2H, 2 × C_{Ar}H), 3.15 (dd, *J* = 12.5, 9.8 Hz, 2H, 2 × C_{Ar}H), 2.98 (ddd, *J* = 12.5, 9.6, 7.3 Hz, 2H, C_{Ar}H), 2.81 (ddd, *J* = 12.3, 9.8, 7.3 Hz, 2H, 2 × C_{Ar}H). – **^{13}C NMR** (101 MHz, DMSO-*d*$_6$) δ/ppm = 167.7 (C_q, 2 × *C*OOH), 141.9 (C_q, 2 × *C*$_{Ar}$), 139.7 (C_q, 2 × *C*$_{Ar}$), 136.1 (+, CH, 2 × *C*$_{Ar}$), 135.9 (+, CH, 2 × *C*$_{Ar}$), 133.3 (+, CH, 2 × *C*$_{Ar}$), 130.7 (C_q, 2 × *C*$_{Ar}$-COOH), 35.3 (–, CH_2, 2 × *C*^{PC}), 33.7 (–, CH_2, 2 × *C*^{PC}). – **IR** (ATR): $\tilde{\nu}$/cm^{-1} = 2925 (w), 1674 (w), 1592 (w), 1556 (w), 1489 (vw), 1422 (w), 1300 (w), 1273 (w), 1203 (w), 1074 (w), 909 (w), 850 (vw), 797 (vw), 759 (vw), 717 (vw), 664 (w), 631 (w), 555 (vw), 518 (w), 426 (vw). – **MS** (70 eV, EI) *m*/*z* (%): 296 (27) [M]$^+$, 278 (100) [M–H_2O]+, 148 (83) [M–$C_9H_8O_2$]$^+$. – **HRMS** (EI, $C_{18}H_{16}O_4$) calc. 296.1049, found 296.1049. The analytical data match those reported in literature.[22]

(*R*$_P$,*S*)-4,12-Bis(4'-*iso*propyloxazolin-2'yl)[2.2]paracyclophane (*R*$_P$,*S*)-4a

Thionyl chloride (1.0 mL) was added to (*R*$_P$,*S*)-4,12-dicarboxy[2.2]paracyclophane (250 mg, 0.840 mmol, 1.00 equiv.) and the resulting mixture was stirred at 100 °C for 90 min. After cooling to room temperature the excess thionyl chloride was removed under vacuum, the final traces were washed with toluene (2 × 2 mL) and removed under vacuum. The resulting crude acetyl chloride was

dissolved in CH_2Cl_2 (5 mL) and cooled to 0 °C. A solution of *S*-Valinol (0.350 g, 3.36 mmol, 4.00 equiv.) and Et_3N (0.54 mL, 0.42 g, 4.20 mmol, 5.00 equiv.) in CH_2Cl_2 (1.0 mL) was added, the reaction mixture was allowed to warm to room temperature and stirred for 24 h. 10 mL of CH_2Cl_2 was then added and the solution was washed with aq. $NaHCO_3$ solution (3.5% w/v, 2 × 10 mL) and brine (20 mL). The organic phase was dried over $MgSO_4$, filtered, concentrated and dried under vacuum to give the crude amide as light brown solid.

The crude amide was dissolved in CH_3CN (5.0 mL), PPh_3 (0.66 g, 2.52 mmol, 3.00 equiv.), CCl_4 (0.770 mL, 1.23 g, 7.98 mmol, 9.50 equiv.) and Et_3N (0.970 mL, 0.760 g, 7.56 mmol, 9.00 equiv.) were added subsequently. After stirring at room temperature overnight, the solvent was removed under reduced pressure, the resulting mixture was dissolved in CH_2Cl_2 and washed with H_2O (2 × 10 mL), the combined organic phase was washed with brine, dried over Na_2SO_4, filtrated and concentrated under vacuum. The crude was purified *via* column chromatography (*c*-Hex/EtOAc = 9:1) to give the title product as a pale yellow solid, 0.150 g, 0.350 mmol, 42%.

$\boldsymbol{R_f}$ = 0.34 (*c*-Hex/EtOAc = 9:1). – ^{1}**H NMR** (400 MHz, $CDCl_3$) δ/ppm = 7.09 (d, *J* = 1.9 Hz, 2H, 2 × $C_{Ar}H$), 6.62 (dd, *J* = 7.9, 1.9 Hz, 2H, 2 × $C_{Ar}H$), 6.54 (d, *J* = 7.8 Hz, 2H, 2 × $C_{Ar}H$), 4.37 (ddd, *J* = 11.2, 9.5, 2.0 Hz, 2H, 2 × CH_{PC}), 4.30 (dd, *J* = 5.8, 2.2 Hz, 2H, 2 × $CH^{5'}$), 4.04 (dd, *J* = 8.6, 6.7 Hz, 2H, 2 × $CH^{4'}$), 4.05–3.92 (m, 2H, 2 × $CH^{5'}$), 3.24–3.16 (m, 2H, 2 × CH_{PC}),
3.16–3.07 (m, 2H, 2 × CH_{PC}), 2.82 (ddd, *J* = 12.6, 10.0, 7.1 Hz, 2H, 2 × CH_{PC}), 1.95 (hept, *J* = 6.7 Hz, 2H, 2 × $CH^{6'}$), 1.20 (d, *J* = 6.7 Hz, 6H, $CH^{7'}$), 1.06 (d, *J* = 6.7 Hz, 6H, $CH^{7'}$). – ^{13}**C NMR** (101 MHz, $CDCl_3$) δ/ppm = 162.9 (C_q, 2 × $C^{2'}$), 141.0 (C_q, 2 × C_{Ar}), 140.1 (C_q, 2 × C_{Ar}), 135.8 (+, CH, 2 × C_{Ar}), 134.8 (+, CH, 2 × C_{Ar}), 132.3 (+, CH, 2 × C_{Ar}), 128.2 (C_q, 2 × C_{Ar}), 73.8 (+, CH, 2 × $C^{4'}$), 69.3 (–, CH_2, 2 × $C^{5'}$), 35.8 (–, CH_2, 2 × C^{PC}), 33.6 (+, CH, 2 × $C^{6'}$), 33.6 (–, CH_2, 2 × C^{PC}), 19.7 (+, CH_3, 2 × $C^{7'}$), 19.2 (+, CH_3, 2 × $C^{7'}$). – **IR** (ATR): $\tilde{\nu}$/cm^{-1} = 2955 (w), 1637 (m), 1590 (w), 1492 (w), 1468 (w), 1429 (w), 1384 (w), 1346 (w), 1303 (w), 1275 (w), 1258 (w), 1191 (w), 1172 (w), 1137 (w), 1115 (w), 1053 (m), 1026 (w), 984 (m), 933 (w), 907 (m), 889 (w), 822 (w), 749 (w), 694 (w), 674 (w), 643 (w), 514 (w), 482 (vw), 389 (vw). – **MS** (FAB, 3-NBA), m/z (%): 431 (100) $[M + H]^+$, 500/488 (9/9) $[C_{14}H_{17}NO_2 + H]^+$. – **HRMS** (FAB, $C_{28}H_{35}O_2N_2$, $[M+H]^+$): calc. 431.2699, found 431.2701.

($\boldsymbol{R_P}$,*S*)-4,12-Bis(4'-*tert*butyloxazolin-2'yl)[2.2]paracyclophane ($\boldsymbol{R_P}$,*S*)-4b

Thionyl chloride (2.0 mL) was added to (R_P)-4,12-dicarboxy[2.2]paracyclophane (0.150 g, 0.510 mmol, 1.00 equiv.), after stirring at room temperature for 10 min, the mixture was heated to 100 °C and stirred at this temperature for 90 min. The excess thionyl chloride was removed by evaporation, the final traces were washed with toluene (2 × 2 mL). After drying under vacuum, the resulting crude acid chloride was dissolved in abs. CH_2Cl_2 (5 mL) and cooled to 0 °C. A solution of (*S*)-(+)-*tert*-leucinol (0.229 g, 2.04 mmol, 4.00 equiv.) and abs. Et_3N (0.260 g, 0.360 mL, 2.55 mmol, 5.00 equiv.) in CH_2Cl_2 (1.0 mL) was added and the reaction mixture allowed to warm to room temperature and stirred for 24 h. Water was then added (10 mL) and extracted with CH_2Cl_2 (3 × 10 mL), the combined organic phase was washed with sat. $NaHCO_3$ solution and brine (20 mL). The organic phase was dried over $MgSO_4$, filtrated, concentrated and dried under vacuum. The crude was purified *via* column chromatography (CH_2Cl_2/MeOH = 98:2 → 95:5) to give the intermediate amide. To a solution of this amide (152 mg, 0.307 mmol, 1.00 equiv.) and PPh_3 (282 mg, 1.08 mmol, 3.50 equiv.) in abs. CH_3CN (8.00 mL) was added triethyl amine (0.385 mL, 280 mg, 2.76 mmol, 9.00 equiv.) and CCl_4 (0.281 mL, 449 mg, 2.92 mmol, 9.50 equiv.) under argon atmosphere. After stirring at room temperature over night, the solvent was removed under vacuum, the resulting crude was dissolved in CH_2Cl_2 and washed with brine, the organic phase was dried over Na_2SO_4, filtrated and concentrated under vacuum. The resulting mixture was purified *via* column chromatography (*c*-Hex/EtOAc = 9:1) to give the title product as colorless solid, 104 mg, 0.227 mmol, 44% over two steps.

$\boldsymbol{R_f}$ = 0.36 (*c*-Hex/EtOAc = 9:1). – ^{1}**H NMR** (400 MHz, $CDCl_3$) δ/ppm = 7.12 (d, *J* = 1.9 Hz, 2H, 2 × $C_{Ar}H$), 6.64 (dd, *J* = 7.8, 1.9 Hz, 2H, 2 × $C_{Ar}H$), 6.55 (d, *J* = 7.8 Hz, 2H, 2 × $C_{Ar}H$), 4.33–4.24 (m, 2H, 2 × $CH^{5'}$), 4.20 (td, *J* = 8.8, 3.7 Hz, 2H, 2 × $CH^{4'}$), 4.17–4.08 (m, 4H, 2 × $CH^{5'}$ + 2 × CH_{PC}), 3.21–3.03 (m, 4H, 4 × CH_{PC}), 2.86–2.68 (m, 2H, 2 × CH_{PC}), 0.99 (s, 18H, $CH^{7'}$). – ^{13}**C NMR** (101 MHz, $CDCl_3$) δ/ppm = 162.9 (C_q, 2 × $C^{2'}$), 141.0 (C_q, 2 × C_{Ar}), 140.2 (C_q, 2 × C_{Ar}), 135.6 (+, CH, 2 × C_{Ar}), 134.7 (+, CH, 2 × C_{Ar}), 132.2 (+,

CH, 2 × C_{Ar}), 128.0 (C_q, 2 × C_{Ar}), 74.2 (+, CH, 2 × $C^{4'}$), 67.7 (–, CH_2, 2 × $C^{5'}$), 36.2 (–, CH_2, 2 × C^{PC}), 34.1 (–, CH_2, 2 × C^{PC}), 34.0 (C_q, 2 × $C^{6'}$), 26.1 (+, CH_3, 6 × $C^{7'}$). – **IR** (ATR): $\tilde{\nu}$/cm^{-1} = 2951 (w), 2866 (w), 1638 (m), 1590 (w), 1477 (w), 1392 (w), 1350 (w), 1333 (w), 1303 (w), 1257 (w), 1191 (w), 1172 (w), 1113 (w), 1067 (w), 1047 (w), 1024 (w), 979 (m), 930 (w), 906 (w), 819 (w), 791 (w), 719 (w), 679 (w), 632 (w), 544 (vw), 513 (w). – **MS** (FAB, 3-NBA), m/z (%): 459 (82) $[M + H]^+$, 230 (75) $[C_{15}H_{19}NO + H]^+$. – **HRMS** (FAB, $C_{30}H_{39}O_2N_2$, $[M+H]^+$): calc. 459.3012, found 459.3011.

(R_P,S)-4,12-Bis(1'-phenyloxazolin-2'yl)[2.2]paracyclophane (R_P,S)-4c

Thionyl chloride (2.0 mL) was added to (R_P)-4,12-dicarboxy[2.2]paracyclophane (0.150 g, 0.510 mmol, 1.00 equiv.), after stirring at room temperature for 10 min, the mixture was heated to 100 °C and stirred under this temperature for 90 min. The excess thionyl chloride was removed by evaporation and the final traces were washed with toluene (2 × 2 mL). After drying under vacuum, the resulting crude acid chloride was dissolved in abs. CH_2Cl_2 (5 mL) and cooled to 0 °C. A solution of (*S*)-(+)-phenylglycinol (0.280 g, 2.04 mmol, 4.00 equiv.) and abs. Et_3N (0.360 mL, 0.260 g, 2.55 mmol, 5.00 equiv.) in CH_2Cl_2 (1 mL) was added and the reaction mixture allowed to warm to room temperature and stirred for 24 h. Water (10 mL) was then added, the water phase was extracted with CH_2Cl_2 (3 × 10 mL) and the combined organic phase was washed with sat. $NaHCO_3$ solution and brine (20 mL). The organic phase was dried over $MgSO_4$, filtered, concentrated and dried in vacuum. The crude was purified *via* column chromatography (CH_2Cl_2/MeOH = 98:2 → 95:5) to give the intermediate amide. To a solution of this amide (200 mg, 0.374 mmol, 1.00 equiv.) and PPh_3 (344 mg, 1.31 mmol, 3.50 equiv.) in abs. 10 mL of CH_3CN was added Et_3N (0.469 mL, 341 mg, 3.37 mmol, 9.00 equiv.) and CCl_4 (0.343 mL, 547 mg, 3.55 mmol, 9.50 equiv.) under argon atmosphere. After stirring at room temperature overnight, the solvent was removed under vacuum, the resulting crude was dissolved in CH_2Cl_2 and washed with brine. The organic phase was dried over Na_2SO_4, filtrated and concentrated under vacuum, the resulting mixture was purified *via* column chromatography (*c*-Hex/EtOAc = 9:1) to give the title product as colorless solid, 177 mg, 0.355 mmol, 95%.

R_f = 0.14 (*c*-Hex/EtOAc = 9:1). – **^{1}H NMR** (500 MHz, $CDCl_3$) δ/ppm = 7.40–7.29 (m, 12H, $CH^{7'+8'+9'}$ + 2 × $C_{Ar}H$), 6.70 (dd, *J* = 7.9, 1.9 Hz, 2H, 2 × $C_{Ar}H$), 6.61 (d, *J* = 7.9 Hz, 2H, 2 × $C_{Ar}H$), 5.47 (dd, *J* = 10.1, 8.2 Hz, 2H, 2 × $CH^{5'}$), 4.65 (dd, *J* = 10.1, 8.2 Hz, 2H, 2 × $CH^{5'}$), 4.44–4.25 (m, 2H, 2 × CH_{PC}), 4.13 (t, *J* = 8.2 Hz, 2H, 2 × $CH^{4'}$), 3.22–3.14 (m, 4H, 4 × CH_{PC}), 2.89–2.79 (m, 2H, 2 × CH_{PC}). – **^{13}C NMR** (126 MHz, $CDCl_3$) δ/ppm = 164.6 (C_q, 2 × $C^{2'}$), 143.0 (C_q, 2 × $C^{6'}$), 141.4 (C_q, 2 × C_{Ar}), 140.3 (C_q, 2 × C_{Ar}), 135.9 (+, CH, 2 × C_{Ar}), 135.1 (+, CH, 2 × C_{Ar}), 132.8 (+, CH, 2 × C_{Ar}), 128.8 (+, CH, 4 × $C^{8'}$), 128.5 (C_q, CH, 2 × C_{Ar}), 127.5 (+, CH, 2 × $C^{9'}$), 126.9 (+, CH, 4 × $C^{7'}$), 73.9 (+, CH, 2 × $C^{4'}$), 70.7 (–, CH_2, 2 × $C^{5'}$), 36.4 (–, CH_2, 2 × C^{PC}), 34.2 (–, CH_2, 2 × C^{PC}). – **IR** (ATR): $\tilde{\nu}$/cm^{-1} = 2922 (w), 1630 (m), 1589 (w), 1493 (w), 1448 (w), 1349 (w), 1296 (w), 1274 (w), 1245 (w), 1191 (w), 1172 (w), 1136 (vw), 1116 (vw), 1050 (w), 986 (w), 961 (w), 927 (w), 902 (w), 887 (w), 823 (w), 750 (w), 697 (w), 639 (w), 523 (w), 388 (vw). – **MS** (FAB, 3-NBA), m/z (%): 499 (100) $[M + H]^+$, 250 (34) $[C_{17}H_{15}NO + H]^+$. – **HRMS** (FAB, $C_{34}H_{31}O_2N_2$, $[M+H]^+$): calc 499.2386, found 499.2386.

Methyl 2-phenyl-2-(phenylamino)acetate (7a)

General procedure GP was followed by adding phenyl-2-diazopropionate (17.6 mg, 1.00 mmol,1.00 equiv) and aniline (11.2 mg, 1.20 mmol, 1.20 equiv) to a suspension of *in situ* generated Cu-(R_p,S)-**4a** catalyst. The product **7a** was obtained *via* flash chromatography (*c*-Hex/EtOAc = 5:1) as colorless solid, 23.6 mg, 0.98 mmol, 98%.

^{1}H NMR (300 MHz, $CDCl_3$) δ/ppm = 7.42 (d, *J* = 7.7 Hz, 2H, 2 × $C_{Ar}H$), 7.27 (qd, *J* = 7.5, 6.4, 2.6 Hz, 3H, 3 × $C_{Ar}H$), 7.04 (t, *J* = 7.9 Hz, 2H, 2 × $C_{Ar}H$), 6.62 (t, *J* = 7.3 Hz, 1H, $C_{Ar}H$), 6.48 (d, *J* = 7.7 Hz, 2H, 2 × $C_{Ar}H$), 5.01 (d, *J* = 5.9 Hz, 1H, C*H*N), 4.88 (s, 1H, N*H*), 3.65 (s, 3H, OCH_3).

The analytical data matches the data reported in literature.[23]

Methyl 2-phenyl-2-(phenylamino)acetate (7b)

General procedure GP was followed by adding phenyl-2-diazopropionate (17.6 mg, 1.00 mmol,1.00 equiv) and aniline (11.2 mg, 1.20 mmol, 1.20 equiv) to a suspension of *in situ* generated Cu-(R_p,*S*)-**4a** catalyst. The product **7b** was obtained *via* flash chromatography (*c*-Hex/EtOAc = 5:1) as colorless solid, 23.6 mg, 0.98 mmol, 98%.

^{1}H NMR (300 MHz, $CDCl_3$) δ/ppm = 7.42 (d, *J* = 7.7 Hz, 2H, 2 × $C_{Ar}H$), 7.27 (qd, *J* = 7.5, 6.4, 2.6 Hz, 3H, 3 × $C_{Ar}H$), 7.04 (t, *J* = 7.9 Hz, 2H, 2 × $C_{Ar}H$), 6.62 (t, *J* = 7.3 Hz, 1H, $C_{Ar}H$), 6.48 (d, *J* = 7.7 Hz, 2H, 2 × $C_{Ar}H$), 5.01 (d, *J* = 5.9 Hz, 1H, C*H*N), 4.88 (s, 1H, N*H*), 3.65 (s, 3H, OCH_3).

The analytical data matches the data reported in literature.[23]

General Procedure (GP): Copper catalyzed N–H insertion

$Cu(MeCN)_4PF_6$ (5 mol%), ligand (6 mol%) and NaBArF (6 mol%) were added into an oven-dried screw vial, evacuated and backfilled with argon three times. After CH_2Cl_2 (1 mL) was injected into the vial, the solution was stirred at 40 °C under argon atmosphere overnight. A solution of *α*-diazopropionates (1.00 equiv.) and aniline (1.20 equiv.) in CH_2Cl_2 (1 mL) was added dropwise, the mixture was stirred at room temperature for 2 h. The resulting mixture was dried under vacuum and purified *via* column chromatography (*c*-Hex/EtOAc = 8:1 or pentane/Et_2O = 5:1) to give the products **7a-j**.

Benzyl phenylalaninate (7c)

General procedure GP was followed by adding benzyl 2-diazopropanoate (19.0 mg, 1.00 mmol, 1.00 equiv) and aniline (11.2 mg, 1.20 mmol, 1.20 equiv) to a suspension of *in situ* generated Cu-(Rp,S)-**4a** catalyst. The product was obtained as a light yellow solid (*c*-Hex/EtOAc = 4:1), 23.9 mg, 0.94 mmol, 94%.

R_f = 0.33 (*c*-Hex/EtOAc = 5:1). – **^{1}H NMR** (300 MHz, $CDCl_3$) δ/ppm = 7.31–7.16 (m, 5H, CH_2*Ph*), 7.15–7.02 (m, 2H, 2 × *$C_{Ar}H$)*, 6.73 (tt, *J* = 7.3, 1.1 Hz, 1H, $C_{Ar}H$), 6.63 (dd, *J* = 8.6, 1.2 Hz, 2H, 2 × $C_{Ar}H$), 5.07 (s, 2H, C*H_2*Ph), 4.14 (q, *J* = 7.0 Hz, 1H, C*H*N), 3.98 (s, 1H, N*H*), 1.42 (d, *J* = 7.0 Hz, 3H, CHC*H_3*).

The analytical data matches the data reported in literature.[24]

Benzyl (2-methoxyphenyl)alaninate (7d)

General procedure GP was followed by adding benzyl 2-diazopropanoate (19.0 mg, 1.00 mmol, 1.00 equiv) and *o*-anisidine (14.8 mg, 1.20 mmol, 1.20 equiv) to a suspension of *in situ* generated Cu-(Rp,S)-**4a** catalyst. The product was obtained *via* column chromatography (*c*-Hex/EtOAc = 5:1) as a light yellow solid, 23.3 mg, 0.82 mmol, 82%.

^{1}H NMR (300 MHz, $CDCl_3$) δ/ppm = 7.40–7.06 (m, 5H, CH_2*Ph*), 6.78–6.66 (m, 2H, 2 × $C_{Ar}H$), 6.62 (ddd, *J* = 8.2, 7.2, 1.6 Hz, 1H, $C_{Ar}H$), 6.44 (dd, *J* = 7.6, 1.6 Hz, 1H, $C_{Ar}H$), 5.07 (s, 2H, CH_2Ph), 4.64 (s, 1H, N*H*), 4.12 (q, *J* = 7.0 Hz, 1H, C*H*N), 3.76 (s, 3H, OC*H_3*), 1.44 (d, *J* = 6.9 Hz, 3H, CHC*H_3*). – **^{13}C NMR** (75 MHz, $CDCl_3$) δ/ppm = 174.5 (C_q, CO_2Bn), 147.2 (C_q, *C_{Ar})*, 136.6 (C_q, *C_{Ar})*, 135.8 (C_q, *C_{Ar})*, 128.6 (+, CH, 2 × *C_{Ar})*, 128.3 (+, CH, *C_{Ar})*, 128.2 (+, CH, 2 × *C_{Ar})*, 121.3 (+, CH, *C_{Ar})*, 117.7 (+, CH, *C_{Ar})*, 110.6 (+, CH, *C_{Ar})*, 109.9 (+, CH, *C_{Ar})*, 66.8 (–, CH_2, CH_2Ph), 55.5(+, CH_3, OCH_3), 52.0(+, CH, CHN), 18.9 (+, CH_3).

The analytical data matches the data reported in literature.[24]

Benzyl (3-methoxyphenyl)alaninate (7e)

General procedure GP was followed by adding benzyl 2-diazopropanoate (19.0 mg, 1.00 mmol, 1.00 equiv) and *m*-anisidine (14.8 mg, 1.20 mmol, 1.20 equiv) to a suspension of *in situ* generated Cu-(Rp,S)-**4a** catalyst. The product was obtained *via* column chromatography (*c*-Hex/EtOAc = 5:1) as light yellow solid, 15.1 mg, 53%.

^{1}H NMR (300 MHz, $CDCl_3$) δ/ppm = 7.39–7.27 (m, 5H, CH_2*Ph*), 7.07 (t, *J* = 8.1 Hz, 1H, C_{Ar}H), 6.32 (dd, *J* = 8.2, 2.3 Hz, 1H, C_{Ar}H), 6.25–6.19 (m, 1H, C_{Ar}H), 6.16 (t, *J* = 2.3 Hz, 1H, C_{Ar}H), 5.16 (s, 2H, C*H*$_2$Ph), 4.19 (q, *J* = 6.9 Hz, 1H, C*H*N), 3.74 (s, 1H, N*H*), 1.48 (d, *J* = 6.9 Hz, 3H, CHC*H*$_3$). – **^{13}C NMR** (75 MHz, $CDCl_3$) δ/ppm = 174.3 (C_q, *C*O_2Bn), 160.8 (C_q, *C$_{Ar}$)*, 147.9 (C_q, *C$_{Ar}$)*, 135.5 (C_q, *C$_{Ar}$)*, 130.1 (+, CH, *C$_{Ar}$)*, 128.5 (+, CH, 2 × *C$_{Ar}$)*, 128.3 (+, CH, *C$_{Ar}$)*, 128.1 (+, CH, 2 × *C$_{Ar}$)*, 106.3 (+, CH, *C$_{Ar}$)*, 103.7 (+, CH, *C$_{Ar}$)*, 99.5 (+, CH, *C$_{Ar}$)*, 66.8 (–, CH_2, *C*H_2Ph), 55.0 (+, CH_3, O*C*H_3), 52.0 (+, CH, *C*HN), 18.8 (+, *C*H_3).

Benzyl (4-methoxyphenyl)alaninate (7f)

General procedure GP was followed by adding benzyl 2-diazopropanoate (19.0 mg, 1.00 mmol, 1.00 equiv) and *p*-anisidine (14.8 mg, 1.20 mmol, 1.20 equiv) to a suspension of *in situ* generated Cu-(Rp,S)-**4a** catalyst. The product was obtained *via* column chromatography (*c*-Hex/EtOAc = 4:1) as light yellow solid, 20.0 mg, 70%.

^{1}H NMR (300 MHz, $CDCl_3$) δ/ppm = 7.38–7.03 (m, 5H, CH_2*Ph*), 6.72–6.61 (m, 2H, 2 × C_{Ar}H), 6.51 (d, *J* = 9.0 Hz, 2H, 2 × C_{Ar}H), 5.06 (s, 2H, C*H*$_2$Ph), 4.04 (q, *J* = 6.9 Hz, 1H, C*H*N), 3.66 (s, 3H, C*H*$_3$). – **^{13}C NMR** (75 MHz, $CDCl_3$) δ/ppm = 175.3 (C_q, CO_2Bn), 153.4 (C_q, *C$_{Ar}$)*, 141.2 89 (C_q, *C$_{Ar}$)*, 136.1 (C_q, *C$_{Ar}$)*, 129.1 (+, CH, 2 × *C$_{Ar}$)*, 128.8 (+, CH, *C$_{Ar}$)*, 128.6 (+, CH, 2 × *C$_{Ar}$)*, 115.7 (+, CH, 2 × *C$_{Ar}$)*, 115.4 (+, CH, 2 × *C$_{Ar}$)*, 67.2 (–, CH_2, CH_2Ph), 56.2 (+, CH_3 OCH_3,), 53.8 (+, CH, *C*HN), 19.5 (+, CH_3).

The analytical data matches the data reported in literature.[25]

Benzyl *o*-tolylalaninate (7g)

General procedure GP was followed by adding benzyl 2-diazopropanoate (19.0 mg, 1.00 mmol, 1.00 equiv) and *o*-toluidine (12.9 mg, 1.20 mmol, 1.20 equiv) to a suspension of *in situ* generated Cu-(Rp,S)-**4a** catalyst. The product was obtained *via* column chromatography (*c*-Hex/EtOAc = 5:1) as colorless solid, 18.3 mg, 0.68 mmol, 68%.

^{1}H NMR (300 MHz, $CDCl_3$) δ/ppm = 7.44–7.27 (m, 5H, CH_2Ph), 7.15–7.05 (m, 2H, Ph), 6.72 (td, *J* = 7.4, 1.2 Hz, 1H, Ph), 6.55 (dd, *J* = 8.4, 1.2 Hz, 1H, Ph), 5.19 (s, 2H, C*H*$_2$Ph), 4.27 (q, *J* = 6.9 Hz, 1H, C*H*CH_3), 4.08 (s, 1H, N*H*), 2.21 (s, 3H, C*H*$_3$), 1.55 (d, *J* = 6.9 Hz, 3H, CHC*H*$_3$). – **^{13}C NMR** (75 MHz, $CDCl_3$) δ/ppm = 174.7 (C_q, *C*O_2Bn), 144.7 (C_q, *C$_{Ar}$)*, 135.7 (C_q, *C$_{Ar}$)*, 130.5 (C_q, *C$_{Ar}$)*, 128.7 (+, CH, 2 × *C$_{Ar}$)*, 128.4 (+, CH, *C$_{Ar}$)*, 128.2 (+, CH, 2 × *C$_{Ar}$)*, 127.2 (+, CH, *C$_{Ar}$)*, 122.8 (+, CH, *C$_{Ar}$)*, 118.0 (+, CH, *C$_{Ar}$)*, 110.5 (+, CH, *C$_{Ar}$)*, 66.9 (–, CH_2, *C*H_2Ph), 52.1 (+, CH, *C*HN), 19.2 (+, *C*H_3), 17.5 (+, *C*H_3).

Benzyl p-tolylalaninate (7h)

General procedure GP was followed by adding benzyl 2-diazopropanoate (19.0 mg, 1.00 mmol, 1.00 equiv) and *p*-toluidine (12.9 mg, 1.20 mmol, 1.20 equiv) to a suspension of *in situ* generated Cu-(S_P,S)/(R_P,S)-**4a** catalyst.The product was obtained *via* column chromatography (*c*-Hex/EtOAc = 4:1) as light yellow solid, 18.8 mg, 0.70 mmol, 70%.

R_f = 0.31 (*c*-Hex/EtOAc = 5:1). – **^{1}H NMR** (300 MHz, $CDCl_3$) δ/ppm = 7.34–7.14 (m, 5H, CH_2*Ph*), 6.89 (d, *J*=8.1 Hz, 2H, 2 × C_{Ar}H), 6.45 (d, *J* = 8.4 Hz, 2H, 2 × C_{Ar}H), 5.06 (s, 2H, C*H*$_2$Ph), 4.09 (q, *J* = 7.0 Hz, 1H, C*H*N), 3.93 (s, 1H, N*H*), 2.16 (s, 3H, C*H*$_3$), 1.39 (d, *J* = 6.9 Hz, 3H, CHC*H*$_3$). – **^{13}C NMR** (75 MHz, $CDCl_3$) δ/ppm = 174.7 (C_q, CO_2Bn), 144.4 (C_q, *C$_{Ar}$)*, 135.7 (C_q, *C$_{Ar}$)*, 129.9 (C_q, *C$_{Ar}$)*, 128.6 (+, CH, 2 × *C$_{Ar}$H)*,

128.4 (+, CH, *C_{Ar}H)*, 128.2 (+, CH, 2 × *C_{Ar}H)*, 127.8 (+, CH, *C_{Ar}H)*, 113.9 (+, CH, *C_{Ar}H)*, 66.8 (–, CH_2, *C*H_2Ph), 52.6 (+, CH, *C*HN), 20.5 (+, *C*H_3), 19.0 (+, *C*H_3).

Phenyl phenylalaninate (7i)

General procedure GP was followed by adding methyl 2-diazo-2-phenylacetate (17.6 mg, 1.00 mmol, 1.00 equiv) and aniline (11.2 mg, 1.20 mmol, 1.20 equiv) to a suspension of *in situ* generated Cu-(Rp,S)-**4a** catalyst. The product was obtained *via* column chromatography (*c*-Hex/EtOAc = 5:1) as light yellow liquid, 16.3 mg, 0.68 mmol, 68%.
^{1}H NMR (300 MHz, $CDCl_3$) δ/ppm = 7.29 (td, *J* = 7.4, 6.8, 1.3 Hz, 2H, 2 × C_{Ar}H), 7.20–7.03 (m, 3H, 3 × C_{Ar}H), 6.98–6.87 (m, 2H, 2 × C_{Ar}H), 6.72 (tt, *J* = 7.3, 1.1 Hz, 1H, C_{Ar}H), 6.64 (dt, *J* = 7.7, 1.1 Hz, 2H, 2 × C_{Ar}H), 4.32 (q, *J* = 7.0 Hz, 1H, C*H*N), 4.12 (s, 1H, N*H*), 1.58 (d, *J* = 6.9 Hz, 3H, CHC*H*$_3$).

***tert*-Butyl phenylalaninate (7j)**

General procedure GP was followed by adding *tert*-Butyl 2-diazopropanoate (15.6 mg, 1.00 mmol, 1.00 equiv) and aniline (11.2 mg, 1.20 mmol, 1.20 equiv) to a suspension of *in situ* generated Cu-(R_p,*S*)-**4a** catalyst. The product was obtained *via* flash chromatography (*c*-Hex/EtOAc = 8:1) as a light yellow liquid, 16.4 mg, 0.74 mmol, 74%.
^{1}H NMR (300 MHz, $CDCl_3$) δ/ppm = 7.21–7.11 (m, 3H, C_{Ar}H), 6.73 (t, *J* = 7.3 Hz, 1H, C_{Ar}H), 6.61 (d, *J* = 7.7 Hz, 2H, C_{Ar}H), 4.02 (q, *J* = 6.9 Hz, 1H, C*H*N), 1.44 (s, 9H, C(C*H*$_3$)$_3$), 1.43 (d, *J* = 6.9 Hz, 3H). – **^{13}C NMR** (75 MHz, $CDCl_3$) δ/ppm = 174.3 (C_q, *C*O_2*t*Bu), 147.3 (C_q, *C_{Ar})*, 129.8 (+, CH, 2 × *C_{Ar})*, 118.6 (+, CH, *C_{Ar})*, 114.0 (+, CH, 2 × *C_{Ar})*, 82.0 (C_q, *C*(CH_3)$_3$), 53.1 (+, CH, *C*HNH), 28.5 (+, CH_3, 3 × *C*H_3), 19.4 (+, CH_3, CH*C*H_3).

The analytical data matches the data reported in literature.[26]

Methyl 2-phenyl-2-(phenylamino)acetate (14)
General procedure GP was followed by adding phenyl-2-diazopropionate (17.6 mg, 1.00 mmol,1.00 equiv) and aniline (11.2 mg, 1.20 mmol, 1.20 equiv) to a suspension of *in situ* generated Cu-(R_p,*S*)-**4a** catalyst. The product **7b** was obtained *via* flash chromatography (*c*-Hex/EtOAc = 5:1) as colorless solid, 23.6 mg, 0.98 mmol, 98%.

^{1}H NMR (300 MHz, $CDCl_3$) δ/ppm = 7.42 (d, *J* = 7.7 Hz, 2H, 2 × C_{Ar}H), 7.27 (qd, *J* = 7.5, 6.4, 2.6 Hz, 3H, 3 × C_{Ar}H), 7.04 (t, *J* = 7.9 Hz, 2H, 2 × C_{Ar}H), 6.62 (t, *J* = 7.3 Hz, 1H, C_{Ar}H), 6.48 (d, *J* = 7.7 Hz, 2H, 2 × C_{Ar}H), 5.01 (d, *J* = 5.9 Hz, 1H, C*H*N), 4.88 (s, 1H, N*H*), 3.65 (s, 3H, OC*H*$_3$).

The analytical data matches the data reported in literature.[23]

Spectra

(R_p,S)-4,12-Bis(4'-*tert*butyloxazolin-2'yl)[2.2]paracyclophane (R_p,S)-4b

1H spectrum

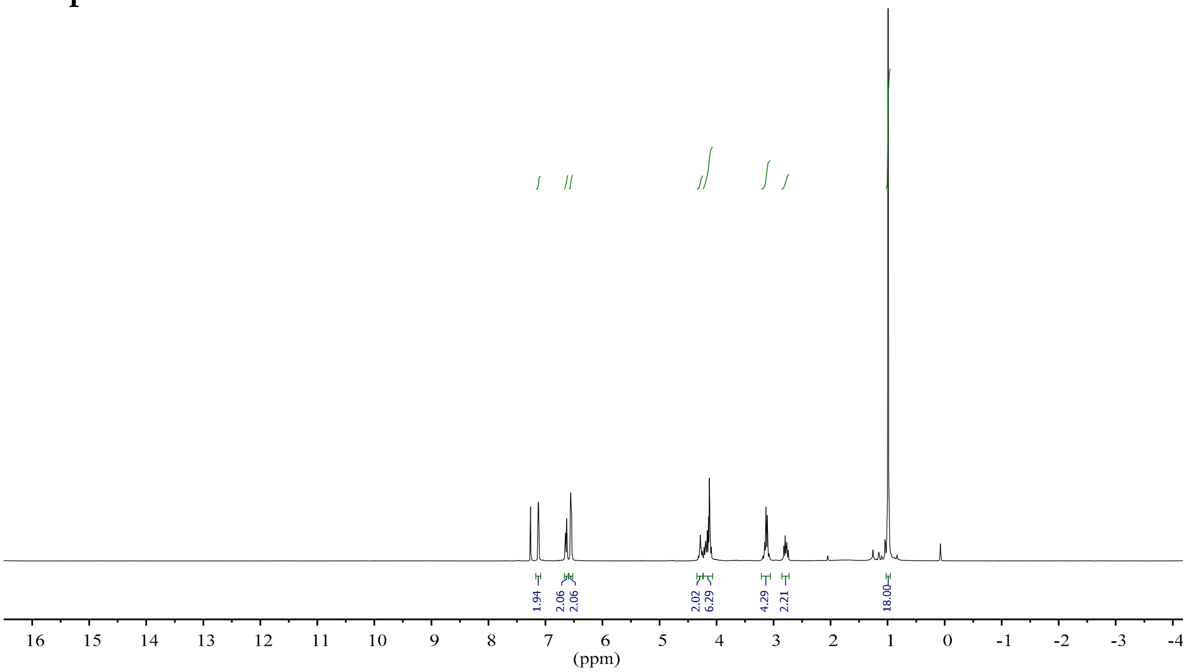

13C spectrum

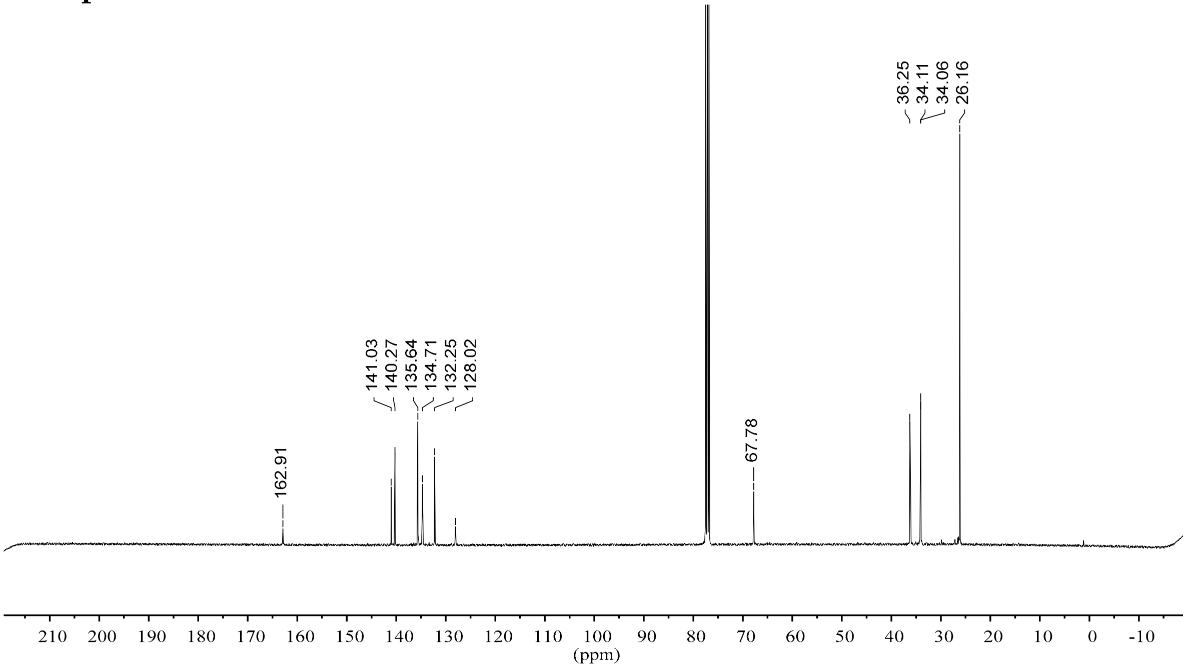

Methyl 3-phenyl-2-(phenylamino)propanoate (7a)
1H spectrum

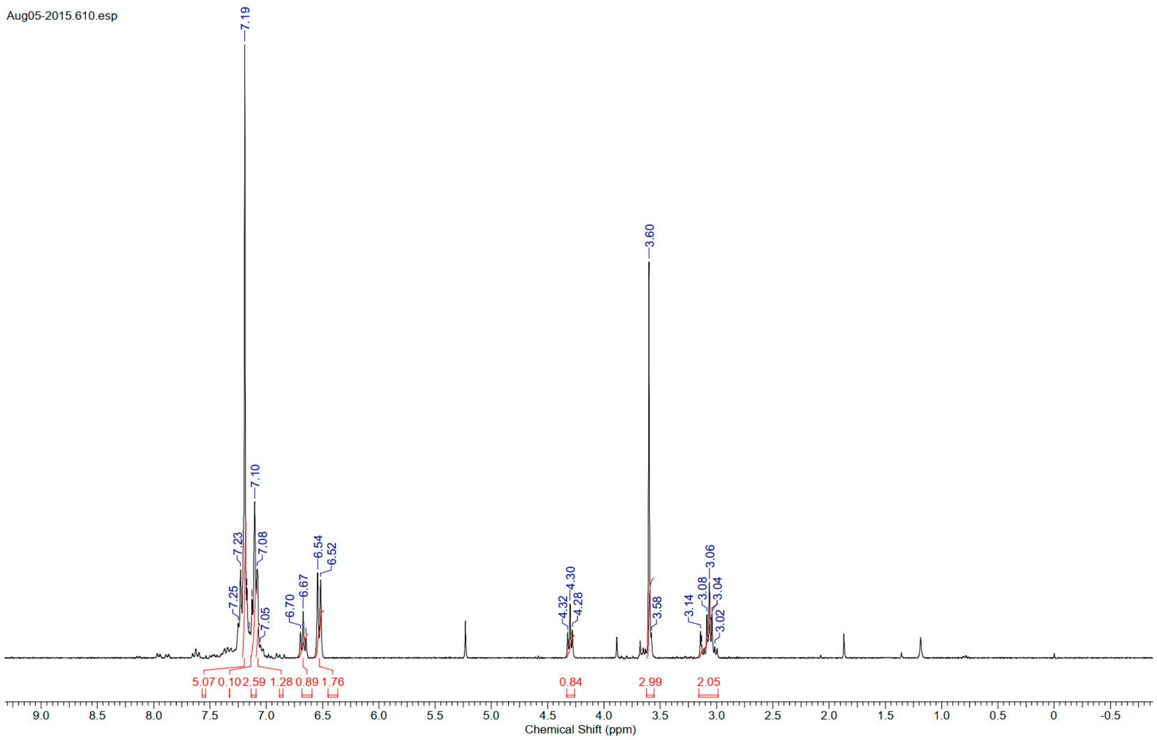

Benzyl (3-methoxyphenyl)alaninate (7e)
1H spectrum

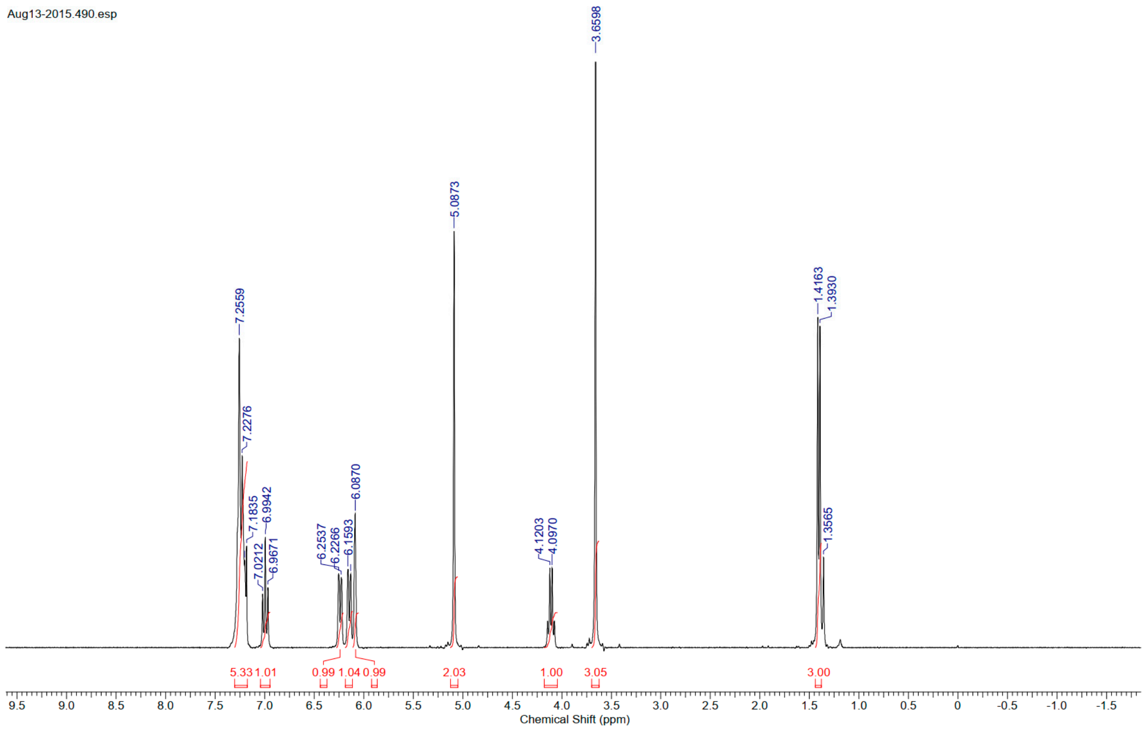

13C spectrum

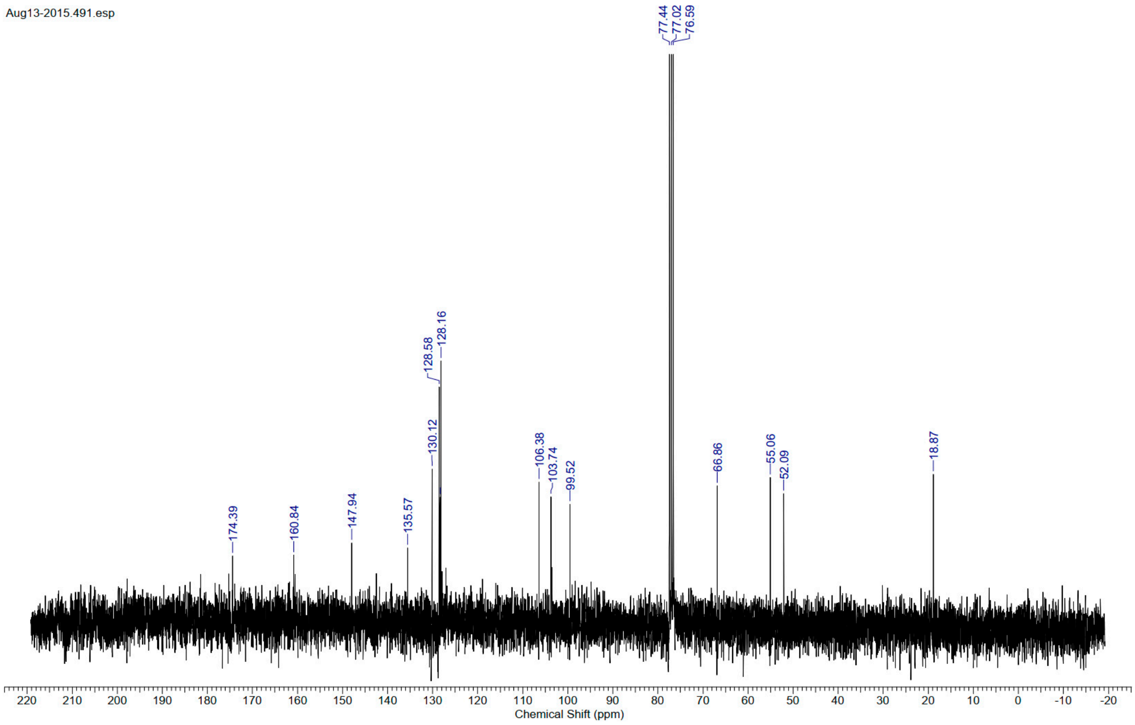

Benzyl *o*-tolylalaninate (7g)

1H spectrum

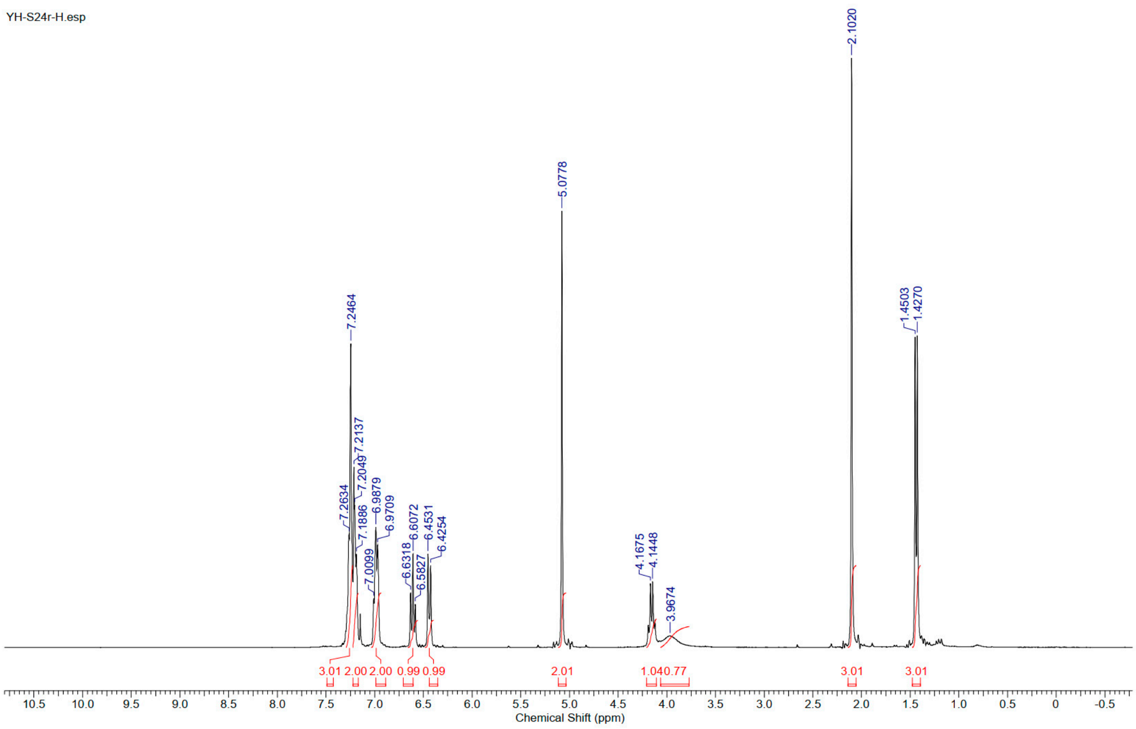

13C spectrum

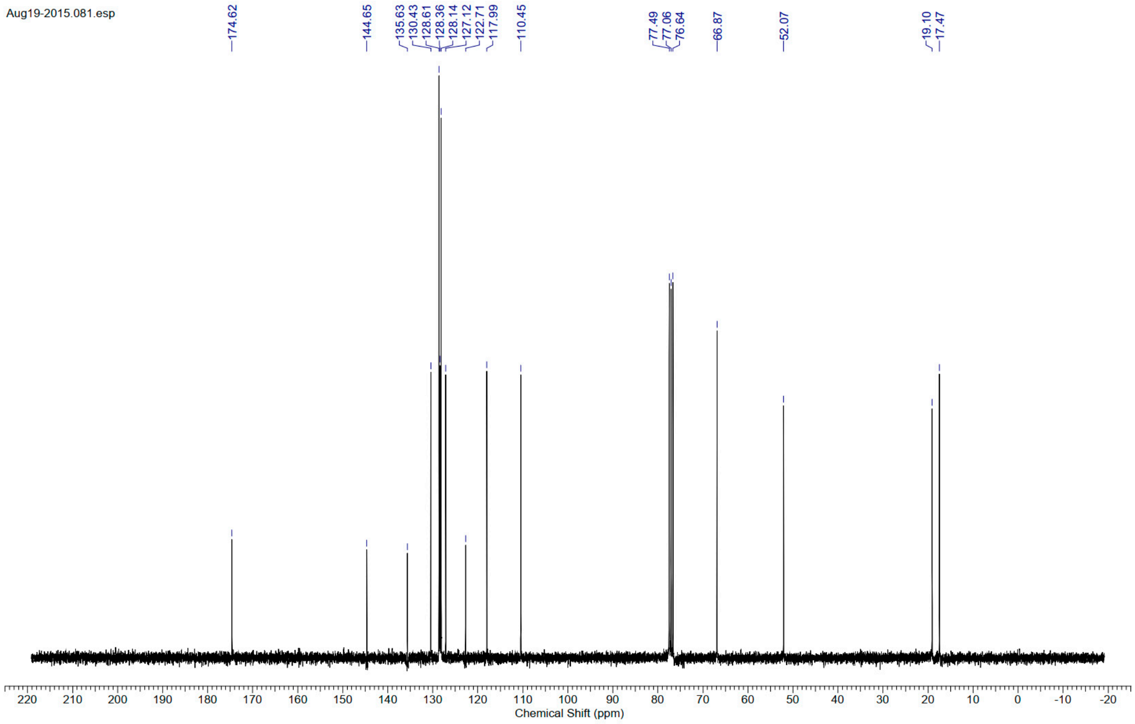

Benzyl p-tolylalaninate (7h)
1H spectrum

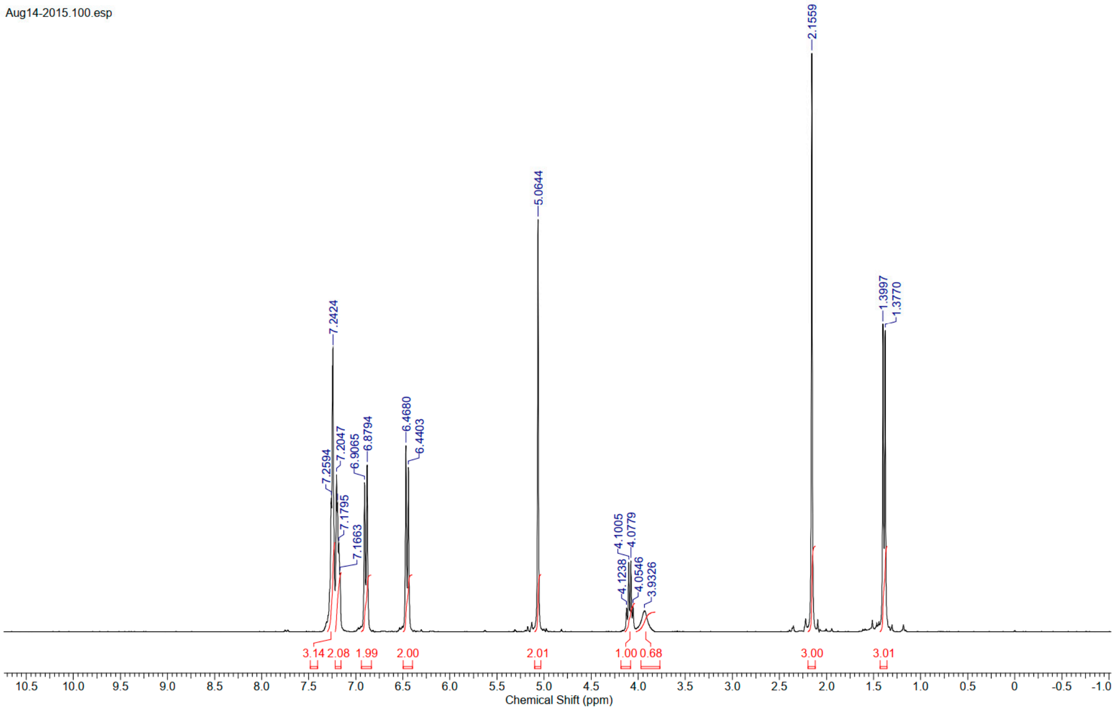

13C spectrum

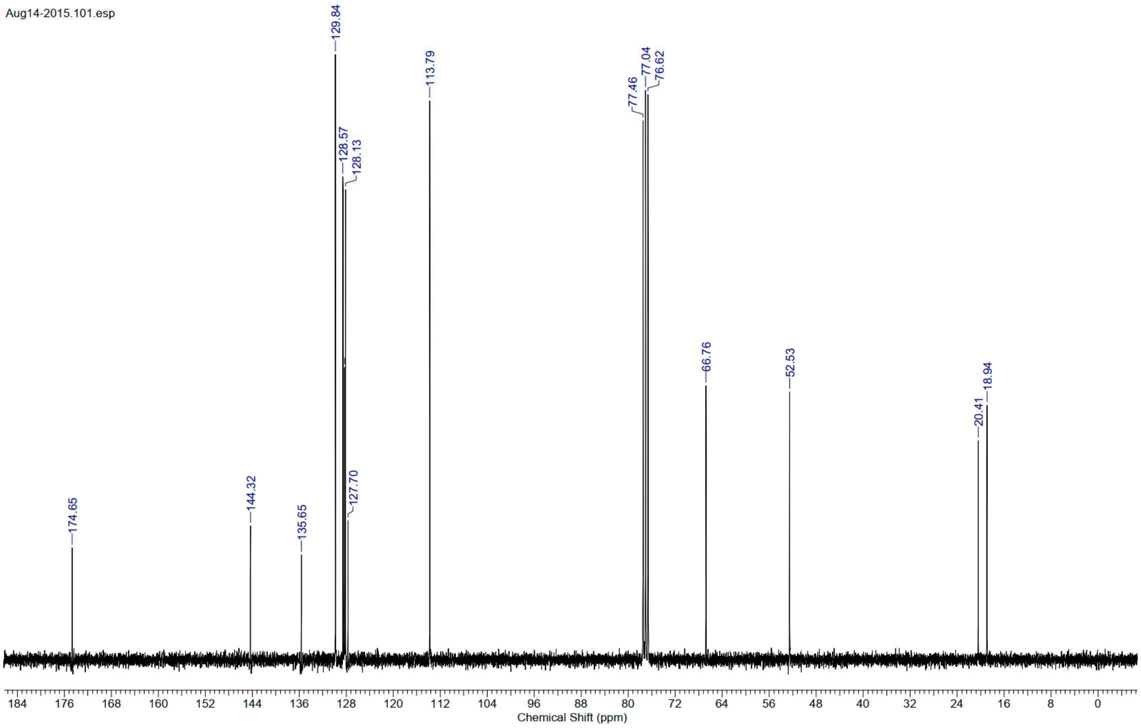

Methyl 2-phenyl-2-(phenylamino)acetate (15)

1H spectrum

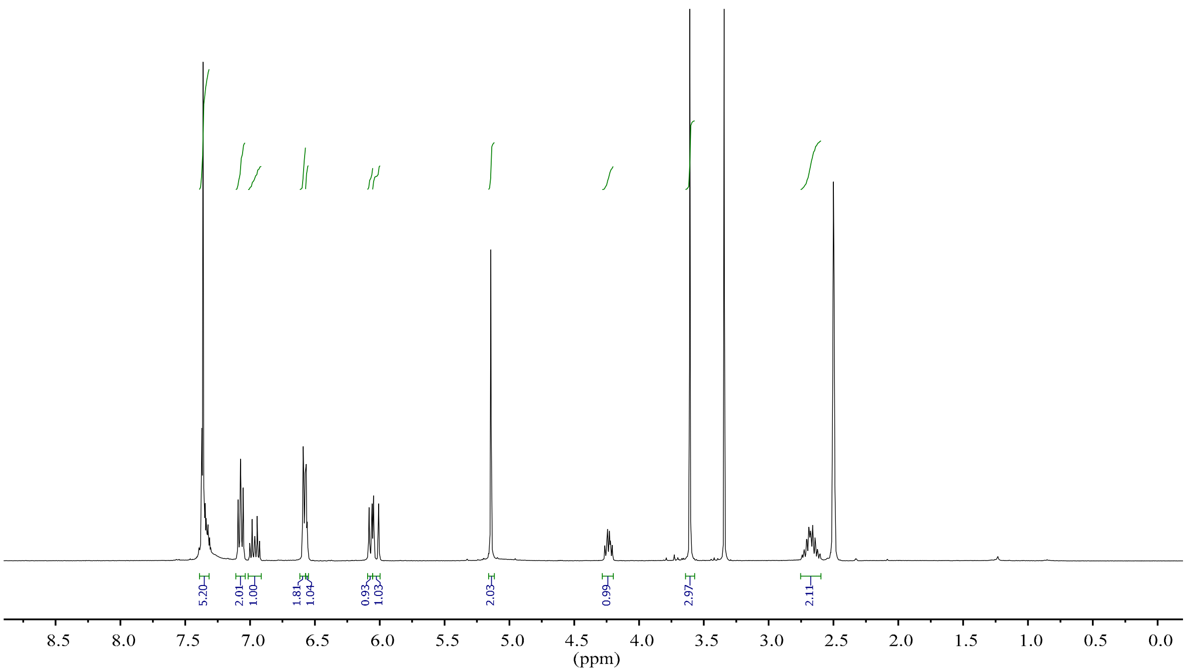

13C spectrum

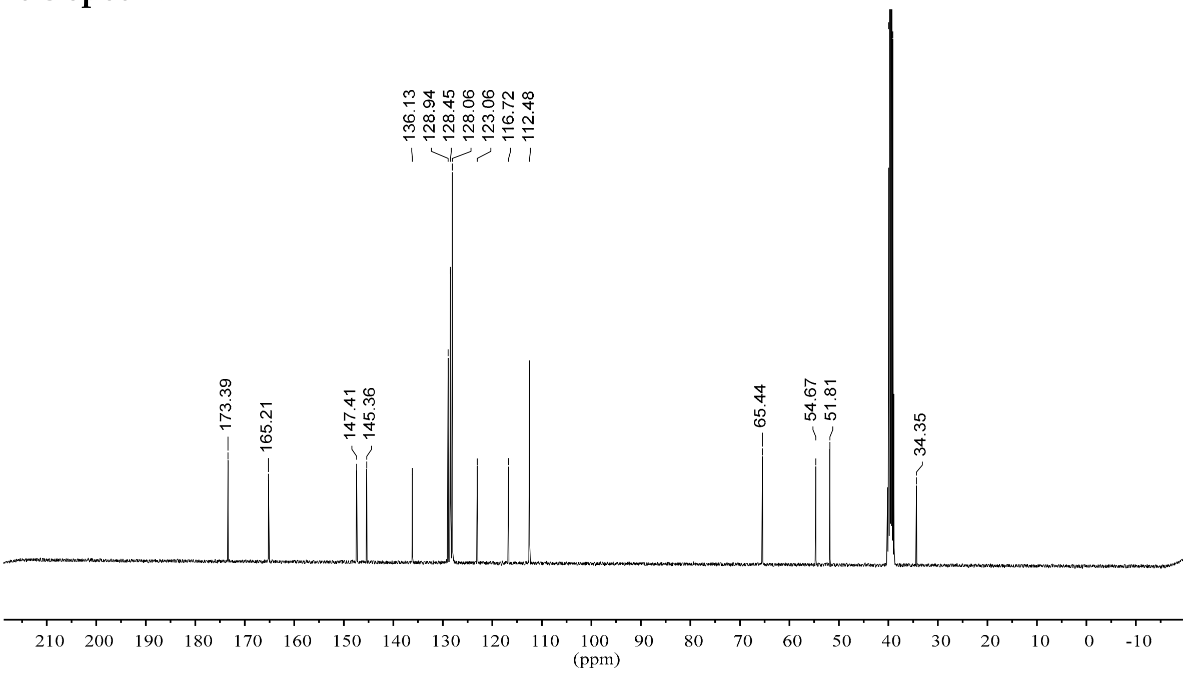

Phenyl phenylalaninate (7i)
1H spectrum

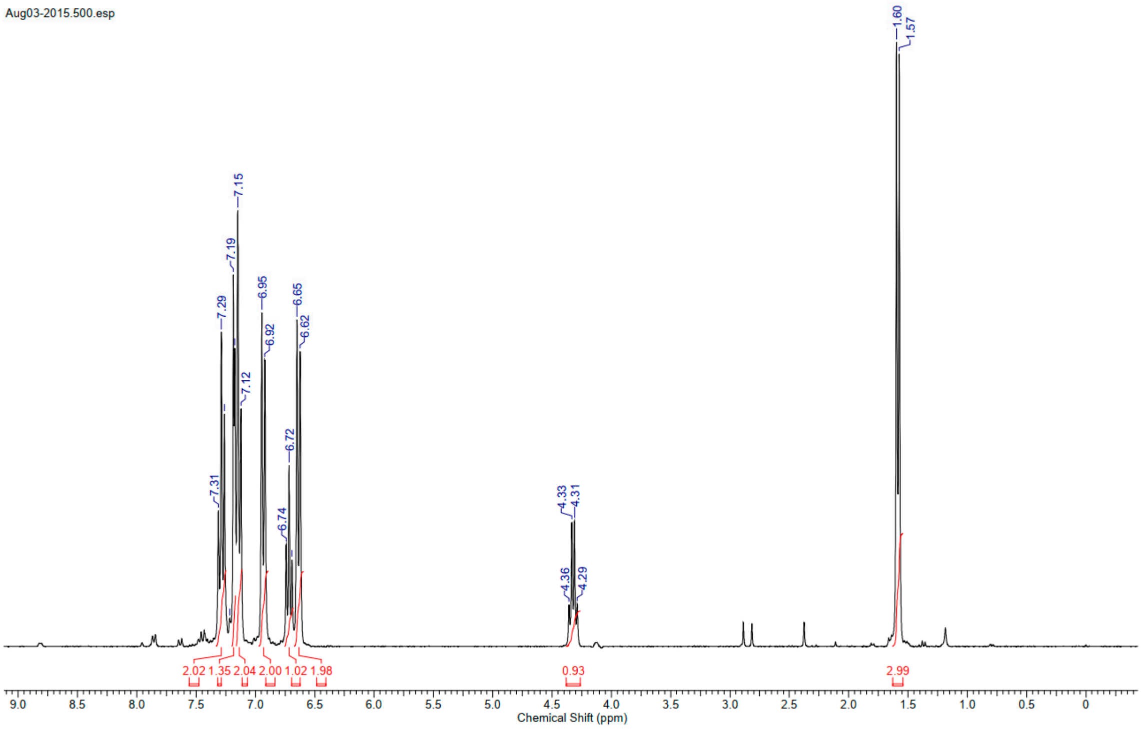

Crystal Structure Determination of 7b

The single-crystal X-ray diffraction study was carried out on a Bruker D8 Venture diffractometer with Photon100 detector at 123(2) K using Cu-Kα radiation (λ = 1.54178 Å. Direct Methods (SHELXS-97) [G. M. Sheldrick, *Acta Crystallogr.* 2008, **A64**, 112-122] were used for structure solution and refinement was carried out using SHELXL-2014 (full-matrix least-squares on F^2) [G. M. Sheldrick, *Acta Crystallogr.* 2015, **C71**, 3-8]. Hydrogen atoms were localized by difference electron density determination and refined using a riding model (H(N) free,). A semi-empirical absorption corrections was and an extinction correction were applied.

7b: colourless crystals, $C_{15}H_{15}NO_2$, M_r = 241.28, crystal size 0.18 × 0.10 × 0.06 mm, monoclinic, space group *C*2/c (No. 15), a = 18.1610(5) Å, b = 5.4218(1) Å, c = 25.4396(7) Å, β = 91.833(1)°, V = 2503.64(11) Å^3, Z = 8, ρ = 1.280 Mg/m^{-3}, μ(Cu-K$_\alpha$) = 0.68 mm^{-1}, F(000) = 1024, T = 123 K, $2\theta_{max}$ = 144.2°, 9009 reflections, of which 2449 were independent (R_{int} = 0.027), 168 parameters, 1 restraint, R_1 = 0.032 (for 2200 I > 2σ(I)), wR_2 = 0.080 (all data), S = 1.07, largest diff. peak / hole = 0.21 / -0.15 e Å^{-3}.

CCDC 1962906 (**7b**) contains the supplementary crystallographic data for this paper. These data can be obtained free of charge from The Cambridge Crystallographic Data Centre via www.ccdc.cam.ac.uk/data_request/cif.

a) SHELXS: G. M. Sheldrick, *Acta Crystallogr.* 2008, **A64**, 112-122.

b) SHELXL-2014 und später: G. M. Sheldrick, *Acta Crystallogr.* 2015, **C71**, 3-8.

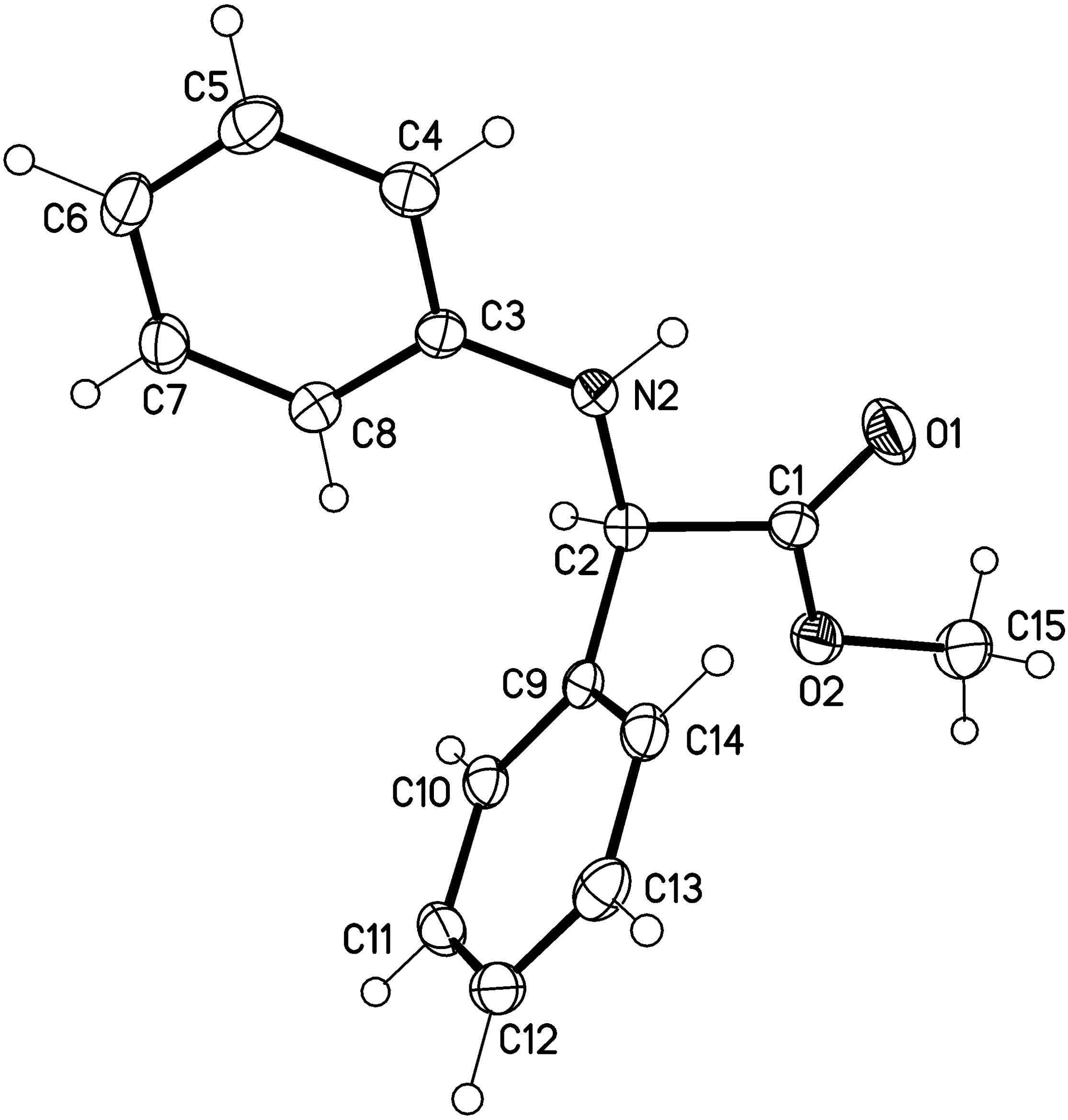

Fig. 1x. Molecular structure of **7b** (displacement parameters are drawn at 50 % probability level).

© 2019 by the authors. Licensee MDPI, Basel, Switzerland. This article is an open access article distributed under the terms and conditions of the Creative Commons Attribution (CC BY) license (http://creativecommons.org/licenses/by/4.0/).
